# Physiological, metabolomic, and transcriptomic reveal metabolic pathway alterations in *Gymnocypris przewalskii* due to cold exposure

**DOI:** 10.1186/s12864-023-09587-9

**Published:** 2023-09-14

**Authors:** Sijia Liu, Fei Tian, Delin Qi, Hongfang Qi, Yang Wang, Shixiao Xu, Kai Zhao

**Affiliations:** 1 Qinghai Provincial Key Laboratory of Animal Ecological Genomics, Key Laboratory of Adaptation and Evolution of Plateau Biota, Northwest Institute of Plateau Biology, Chinese Academy of Sciences, No. 23 Xinning Road, Xining, 810008 Qinghai China; 2 State Key Laboratory of Plateau Ecology and Agriculture, Qinghai University, Xining, Qinghai China; 3 Qinghai Provincial Key Laboratory of Breeding and Protection of Gymnocypris Przewalskii, Qinghai Naked Carp Rescue Center, Xining, Qinghai China

**Keywords:** Cold adaptation, *Gymnocypris przewalskii*, Transcriptome, Metabolome

## Abstract

Teleost fish have evolved various adaptations that allow them to tolerate cold water conditions. However, the underlying mechanism of this adaptation is poorly understood in Tibetan Plateau fish. RNA-seq combined with liquid chromatography‒mass spectrometry (LC‒MS/MS) metabolomics was used to investigate the physiological responses of a Tibetan Plateau-specific teleost, *Gymnocypris przewalskii*, under cold conditions. The 8-month *G. przewalskii* juvenile fish were exposed to cold (4 ℃, cold acclimation, CA) and warm (17 ℃, normal temperature, NT) temperature water for 15 days. Then, the transcript profiles of eight tissues, including the brain, gill, heart, intestine, hepatopancreas, kidney, muscle, and skin, were evaluated by transcriptome sequencing. The metabolites of the intestine, hepatopancreas, and muscle were identified by LC‒MS/MS. A total of 5,745 differentially expressed genes (DEGs) were obtained in the CA group. The key DEGs were annotated using Gene Ontology and Kyoto Encyclopedia of Genes and Genomes analysis. The DEGs from the eight tissues were significantly enriched in spliceosome pathways, indicating that activated alternative splicing is a critical biological process that occurs in the tissues to help fish cope with cold stress. Additionally, 82, 97, and 66 differentially expressed metabolites were identified in the intestine, hepatopancreas, and muscle, respectively. Glutathione metabolism was the only overlapping significant pathway between the transcriptome and metabolome analyses in these three tissues, indicating that an activated antioxidative process was triggered during cold stress. In combination with the multitissue transcriptome and metabolome, we established a physiology-gene‒metabolite interaction network related to energy metabolism during cold stress and found that gluconeogenesis and long-chain fatty acid metabolism played critical roles in glucose homeostasis and energy supply.

**Supplementary Information:**

The online version contains supplementary material available at 10.1186/s12864-023-09587-9.

## Introduction

The Tibetan Plateau is known as “the world’s third pole” with extremely low temperatures. Schizothoracins are endemic to the Qinghai-Tibet Plateau (QTP) and are the main wild fish resource [1]. Substantial climate changes have become more prominent in the QTP [2–4], which could cause strong pressure for Schizothorax fish to adapt to abnormally low temperatures. However, the mechanism underlying the response of plateau fish to low temperatures and the fluctuations in metabolites during cold adaptation are yet unknown. The Tibetan naked carp, named *Gymnocypris przewalskii*, is a rare cyprinid fish species primarily distributed in the ecologically fragile Lake Qinghai and several inflowing freshwater streams and rivers in Qinghai Province, China. Qinghai Lake is extensive, with a maximum water depth of 30 m [5]. Water temperatures are affected by season, air temperature, solar radiation, snowmelt runoff, and water depth. [6]. For example, the average surface water temperature could reach 17.3 °C in summer [7], while the water temperature at the bottom of the lake holds steady at 4–6 °C [8]. In addition, water has more considerable temperature differences due to the plateau climate. Therefore, the fish could experience rapid temperature changes in the ecological environment due to water layer migration or diurnal and seasonal temperature differences [5]. Tong et al. identified several potential expansive gene families in *G. przewalskii* related to energy metabolism, transport, and developmental functions that possibly underlie the adaptation to extremely severe environments [9]. Nonetheless, understanding of the cold tolerance mechanisms of this species is still limited.

Fish are ectothermic (heterothermic) animals that lack a heat-radiating mechanism to maintain a constant body temperature, unlike homeothermic animals. Environmental temperature is an important factor controlling fish distribution, behaviour, and physiological responses since their body temperature is affected by ambient water temperature [10]. At the molecular level, low temperatures decrease the enzymatic reaction rates while reducing the diffusion and transport of biomolecules and slowing protein denaturation and degradation [11]. Low temperatures also inhibit transcription and translation, disrupt cellular cytoskeletal elements, alter membrane penetration ability, and affect energy production in cells [12]. Teleosts possess various strategies to cope with cold stress [13]. Some polar zone fish have antifreeze proteins (AFPs), antifreeze glycoproteins (AFGPs) [14, 15], and tubulin that can be synthesized at low temperatures and when haemoglobin levels are low [16, 17]. Migratory fish can travel through a wide range of areas and move to areas with suitable water temperatures to maintain homeostasis for continued physiological functions [18]. However, fish with poor swimming ability or confined to lakes and rivers are forced to adapt to climate variation and change. Thus, a physiological cascade of acclimation strategies must be activated in fish species to maintain cellular homeostasis during temperature stress [12, 19]. Several fish species reduce their physiological activity by decreasing their routine metabolic output under low-temperature conditions [20]. This metabolic adjustment allows the organism to sustain a minimum necessary level of aerobic energy production to survive hypothermal stress. The energy supply is allocated primarily to maintain essential physiological functions [21, 22].

Despite reducing the overall metabolic rate under low-temperature conditions, cold-adapted fish still require sufficient energy to fulfil the routine energy demands of critical organs and tissues. Thus, fish may have evolved compensatory mechanisms of metabolic regulation that allow critical organs and tissues to maintain normal physiological functions under cold conditions. For example, the activities of several key enzymes, such as lactate dehydrogenase (LDH) and aspartate aminotransferase (AST), are significantly increased at low temperatures and are involved in metabolic processes, as shown in *Epinephelus coioides* [23], *Takifugu obscurus* [24], and *Cyprinus carpio* [25]. Unlike mammals, carbohydrate conversion and utilization are genetically defective in fish, and the lipid derivatives involved in energy metabolism or membrane structure modification are irreplaceable in cold adaptation [26]. Temperate fish, such as *Oncorhynchus mykiss* [27], *Lates calcarifer* [28], and *C. carpio* [29], may convert their energy storage/utilization preference and modify the lipids to maintain the fluidity of cell membranes and reduce energy consumption when the ambient temperature drops seasonally. Metabolism is regulated by gene expression modulation. These gene-coding products are directly involved in metabolic processes or indirectly affect metabolic pathways. Typically, the genes, metabolites, and metabolic regulatory mechanisms involved in cold adaptation are multifarious among teleosts due to their genetic diversity [30, 31].

RNA-seq provides a “snapshot” profile of gene transcription in a certain space–time condition of the biological system [32]. Zebrafish (*Danio rerio*), an aquatic model animal, is the most studied species in the transcriptional mechanisms of cold adaptation. Long et al. [33] revealed that several differentially expressed genes (DEGs) were enriched in RNA processing, protein transport, the mTOR signalling pathway, and the P53 signalling pathway when zebrafish embryos coped with cold stress. Hu et al. [34] detected transcriptional changes in eight zebrafish tissues exposed to cold temperatures and found that different tissues respond to cold stress with distinct strategies, indicating diversified cold-responsive gene expression in zebrafish. Transcriptome analysis of cold adaptation has also been applied to many commercial fishes, such as *Oreochromis niloticus* [35], *Larimichthys crocea* [36], and *Paralichthys olivaceus* [37].

Metabolomics provides a high-throughput metabolite identification strategy in a biological system and is considered an effective tool for detecting the changes in metabolites in response to environmental stressors [38]. Metabolomics techniques have been applied in a few innovative studies to explore the cold stress response in some species of fish, such as *Sparus aurata* [39], *Symphysodon aequifasciatus* [40], and *Sebastes schlegelii* [41]. Metabolomics integrated with other omics datasets provides a myriad of information on the biological mechanisms that has been applied in studies of low-temperature response mechanisms in *Takifugu fasciatus* [42] and *Nibea albiflora* [43].

Herein, 8-month-old juvenile *G. przewalskii* were used as the study model to elucidate the metabolite and transcription profiles in multiple tissues after a 15-day cold adaptation period. Next, by integrating the metabolomic and transcriptomic data, we obtained a comprehensive landscape of the coordination network between metabolites and transcripts involved in the cold adaptation of Tibetan Plateau fish.

## Materials and methods

### Fish

**Fig. 1:**
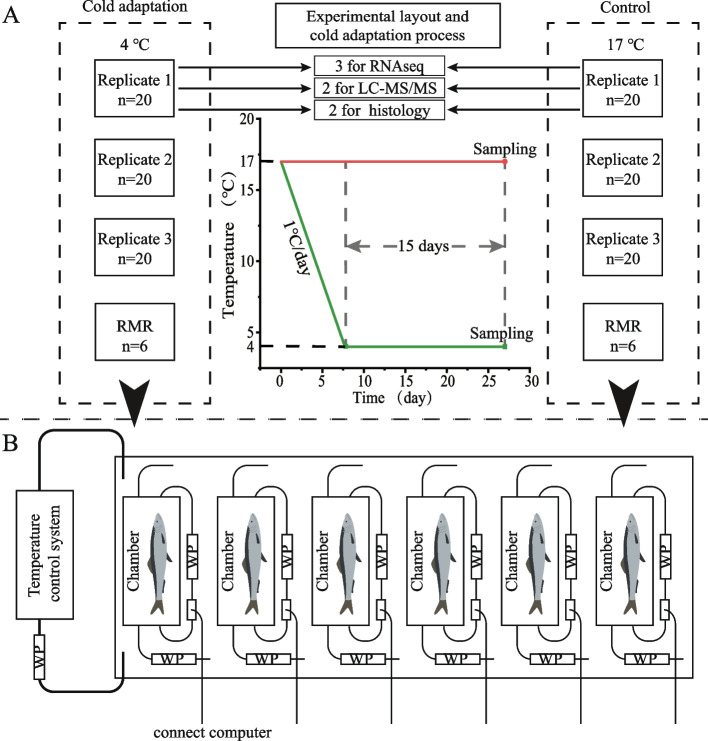
Experimental design and respiratory metabolism detection system. **A** The experiment consisted of two treatments: 4 °C and 17 °C, where *G. przewalskii* was maintained for 15 days. For each treatment, 20 specimens were maintained in three independent replicate tanks (1, 2, and 3). On sampling day (day 15), three fish per replicate tank (*n* = 3) were randomly sampled for RNAseq, two fish per replicate tank (*n* = 2) were randomly sampled for LC–MS/MS, and two fish per replicate tank (*n* = 2) were randomly sampled for histology. **B** A schematic plot of the respiratory metabolism detection system. In each test group (4 °C and 17 °C), six fish were selected randomly for routine metabolism rate (RMR) detection. Individual fish was placed in a 250 mL chamber, submerged in a 100 L outer tank supplied with fresh water at 4 °C or 17 °C. Each chamber was connected with an internal recirculation water pump (WP) and a flush WP. Chamber oxygen partial pressure (pO2) was measured with an OXY-4mini (PreSens, Regensburg, Germany) fiber optic O2 transmitter, connected to a computer. A temperature control system was connected to the outer tank and the water temperature controlled at a desired value

Juvenile *G. przewalskii*, aged 8 months, approximately 6 g, were obtained from Qinghai Naked Carp Rescue Center, China. Fish were kept at the Northwest Institute of Plateau Biology, CAS, China, in a tank (1000 L) filled with circulating fresh water for 30 days at 17 ± 0.5 °C. Then, 120 fish (6.43 ± 0.91 g) were randomly distributed into six (2 treatments × 3 replications) 100 L tanks (*n* = 20/tank) with a density of 0.2 fish perlite (Fig. 1A). Subsequently, 12 fish (6.08 ± 1.07 g) were randomly selected for routine metabolism rate (RMR) detection. For each temperature treatment, replicate tanks were independently equipped with a recirculating water supply and a temperature control system adjusted to 17 ± 0.5 °C. Each tank was equipped with continuous aeration and maintained at a 12:12 light–dark regime. Fish were acclimated to the abovementioned temperature and photoperiod for 7 days before the experiment. Fish were fed (twice a day, 09:00 and 16:00) with commercial pellets containing 43% protein (Hanye Biotechnology Co., Ltd, China) to satiation. After 7 days, fish were progressively exposed to two experimental temperatures: 4 °C and 17 °C in freshwater. The changes in temperatures progressed by 1 °C each day until the desired temperature was reached. Each experimental group was maintained at the desired temperature for 15 days. The required experimental temperatures were maintained with thermostatic heating and cooling systems (EHEIM, Germany; HAILI, China). Temperature, pH (7.80–8.30), and dissolved oxygen (OD > 7.5 and < 12.3) were monitored daily (Professional Series Sensor Installation Instructions, YSI Inc., USA). The uneaten food and fish faeces were removed daily. Freshwater with the desired temperature was exchanged at a rate of 40% twice a day to minimize the build-up of nitrates and nitrites and maintain water quality. No difference in food, oxygen supply, or illumination time was observed between the cold acclimation groups and controls during the treatment.

### Tissue sampling

A total of 9 fish were randomly sampled from each replicate tank of each temperature treatment (*n* = 27) on Day 15. Before sampling, fish were terminally anaesthetized with a lethal dose (500 mg/L) of MS-222 (Zhonghong Biological Engineering Co., Ltd, China) and blotted dry before the body weight and length were measured. Approximately > 200 μL blood was withdrawn from the caudal vein (with 1.5 mg/mL EDTA anticoagulant) to measure the blood glucose (Glu) and triglyceride (TG) levels using an automatic biochemical analyser (ADVIA 2400, Siemens, Japan). Subsequently, fish were dissected on ice, and the brains, hearts, hepatopancreas, intestines, gills, kidneys, muscles (just above the lateral line), and skins were removed, flash-frozen in liquid nitrogen, and stored at -80 °C for RNA-seq and liquid chromatography‒mass spectrometry (LC‒MS/MS) metabolite detection. In addition, on Day 15, two additional fish were sampled to collect the hepatopancreas and muscle samples for histological study.

### Evaluation of performance parameters

The length and weight of all fish were measured at the beginning and end of the experiment. The mean individual weight of each experimental unit was determined by dividing the bulk weight by the number of fish. The following performance parameters and somatic indices were evaluated:

- Condition factor (CF, %) = 100 × live weight/(fish body length)^3^;
- Weight gain (WG, %) = 100 × (final weight − initial weight)/initial weight;
- Hepatosomatic index (HSI, %) = 100 × liver weight/fish body weight;
- Intestinal somatic index (ISI, %) = 100 × intestine weight/fish body weight.

### RNA-seq

#### Total RNA extraction, library construction, and high-throughput sequencing

An equivalent of 50 mg tissue sample was collected from the brain, heart, gill, hepatopancreas, kidney, intestine, muscle, and skin using sterile scissors and forceps. Total RNA was extracted using a TRIzol reagent kit (Invitrogen, Carlsbad, CA, USA) according to the manufacturer’s protocol. RNA quality was assessed on an Agilent 2100 Bioanalyzer (Agilent Technologies, Palo Alto, CA, USA). The RNA integrity was confirmed using RNase-free agarose gel electrophoresis by intact 18S and 28S ribosomal bands. Library construction and sequencing were carried out at Gene Denovo Biotechnology Co. (Guangzhou, China) using a detailed procedure. The mRNA was enriched using oligo (dT) beads and cleaved into short fragments using buffer [44]. Then, the fragments were reverse-transcribed into cDNA with random primers [44]. Subsequently, second-strand cDNA was synthesized from first-strand cDNA by DNA polymerase I, RNase H, dNTP, and buffer [44, 45]. The purified cDNA fragments were obtained using a QiaQuick PCR extraction kit (Qiagen, Venlo, The Netherlands) [44]. Following end repair, poly(A) was added to the fragments and ligated to the adapters [44, 45]. The cDNA fragments were size-selected by agarose gel electrophoresis and enriched by PCR to be sequenced by the Illumina HiSeq™ 2500 platform [44, 45]. The raw sequences have been deposited in the NCBI Sequence Read Archive (SRA) under BioProject PRJNA843344.

#### Read preprocessing and alignment with the reference genome

Raw data were filtered by fastp (version 0.18.0) [46] to obtain high-quality clean reads. The parameters were as follows: (1) reads containing adapters were removed; (2) reads containing > 10% unknown nucleotides (N) were removed; and (3) low-quality reads containing > 50% low-quality (Q-value ≤ 20) bases were removed.

An index of the *G. przewalskii* reference genome (available in the CNGB database under project number CNP0002001) was constructed, and paired-end clean reads were mapped to the reference genome using HISAT (v2. 2.4) [47] with “rna strandness RF” and other parameters set as default. The obtained SAM files were converted to BAM files and sorted using SAMtools (v1.9) [48]. The sorted BAM files were assembled separately using StringTie (v1.3.4d) [49] (Pertea et al., 2015) with default settings. Subsequently, the assemblies of each sample were merged into a single GTF file. The transcript sequences were extracted from the reference genome using gffread (v0.9.12). (https://github.com/gpertea/gffread).

#### Quantification and DEG analysis

The mapped reads of each sample were assembled using StringTie in a reference-based approach. For each transcription region, the fragment per kilobase of transcript per million mapped reads (FPKM) for each transcription region was calculated to quantify its expression and variations using StringTie. To identify the genes regulated by cold stress, three samples of the same tissue type from CA or NT conditions were regarded as biological replicates. A total of 16 groups, including brain, gill, heart, hepatopancreas, intestine, kidney, muscle, and skin from fish exposed to CA and NT conditions, containing three samples in each group, were included in the DEG analyses. The raw reads and count datasets were analysed using DESeq2 [50] to identify the DEGs between 17 °C and 4 °C of each tissue. Genes with a false discovery rate (FDR) < 0.05 and absolute fold-change ≥ 2 were considered DEGs.

#### Enrichment analyses and functional classification of DEGs

*G. przewalskii* genes combined with new genes identified by transcripts in this study were used to build an OrgDb database by the AnnotationForge package (https://bioconductor.org/packages/release/bioc/html/AnnotationForge.html). The DEGs were used for Gene Ontology (GO) enrichment [51] and Kyoto Encyclopedia of Genes and Genomes (KEGG) [52] pathway analyses by the clusterProfiler package using the *G. przewalskii* OrgDb database.

### Quantitative real-time PCR (qRT‒PCR)

qRT‒PCR analysis was performed in the same samples of RNAseq to validate the results of RNAseq. Primers for qRT‒PCR were designed using the Sangon Biotech web server (https://www.sangon.com/) and oligo7 software [53] based on the open reading frames (ORFs) of the tested genes. The sequence IDs, gene names, primer pairs, and lengths of amplicons are listed in Supplementary Table S4. The relative mRNA expression level was normalized against that of the *β-actin* gene and elongation Factor 1α (*ef-1α*) gene and calculated using the comparative threshold cycle method [54]. The generation of single-cDNA PCR products was confirmed before qRT‒PCR. The mRNA expression levels were determined by qRT‒PCR on a LightCycler® 480 thermocycler (Roche, Germany) in a reaction volume of 20 μL using LightCycler® 480 SYBR Green I Master Mix, following the manufacturer’s protocol. The reaction was conducted at 95 °C for 5 min and 45 cycles of 95 °C for 15 s and 60 °C for 45 s. The relative expression of the target genes was calculated using the 2^−△△Ct^ method [54]. All amplification reactions were carried out in triplicate, and a nontemplate control was also included in each run.

### LC‒MS/MS analysis

The metabolite content (widely targeted metabolome) in the hepatopancreas intestine and skeletal muscle was quantified using liquid chromatography‒mass spectrometry (LC‒MS) at Wuhan Metware Biotechnology Co., Ltd. (Wuhan, China), as described by Chen et al. [55] with minor modifications (Supplementary File 1).

### Histology

At the end of the experiment (Day 15), two fish per tank were sampled for histological appraisal of the trial (*n* = 6). The intestine, hepatopancreas, and muscle were sampled, and a standard 3–5 mm section was dissected from the central portion of the organ. Tissue samples were fixed in 4% paraformaldehyde at room temperature and embedded in wax blocks or frozen using a standard protocol. Approximately 2–3 μm sections were sliced on a motorized rotary microtome machine. The sections were dried and stained with haematoxylin and eosin (HE) or oil red O. The stained sections were examined using a Zeiss Imager. A2 microscope (Zeiss, Germany), and digital images of representative slides were captured using a Micropublisher 6 CCD (QINAQING, USA).

### Respirometry

Currently, the RMRs of fish in both CA and NT conditions were measured during the test using stop-flow respirometry, following previously described protocols that are well-established [55, 56]. RMR was quantified on an automatic intermittent flow-through respirometer using a static respirometry system (Loligo Systems, Toldboden, Denmark). A total of 12 fish (6.08 ± 1.07 g) mentioned in 2.1 were equatorially assigned to one cold adaptation group (4 °C, CA) and another control group (17 °C, NT). Then, the fish were placed in six 250 mL chambers (one per chamber) submerged in two 100 L outer tanks (three chambers per tank) supplied with fresh water under the same conditions. The cold adaptation experimental system and processes were carried out as described in 2.1. The chamber oxygen partial pressure (pO_2_) was measured with an OXY-4 mini (PreSens, Regensburg, Germany) fibre optic O_2_ transmitter and recorded using AutoResp4™ software. The experimental apparatus is shown in Fig. 1B. Mass-specific oxygen consumption (MO_2_) was derived from the decrease in chamber pO_2_ during a 5-min measuring period. Chambers were periodically flushed for 4 min, followed by a closed 1-min wait period. MO_2_ measurements during the following 15 days were used to estimate RMR.

### Statistical analysis

The results are presented as the mean ± standard deviation (SD) in the tables and graphs, and *P* < 0.05 indicated a statistically significant difference. All data were checked for normality and homoscedasticity with Shapiro–Wilk’s normality test and Levene’s test. In the case of nonnormal distribution data and when the homoscedasticity assumption was not given, data were log-transformed. Significant differences between means were evaluated using Tukey’s HSD test.

## Results

### Growth performance and physiological and biochemical indices

**Fig. 2:**
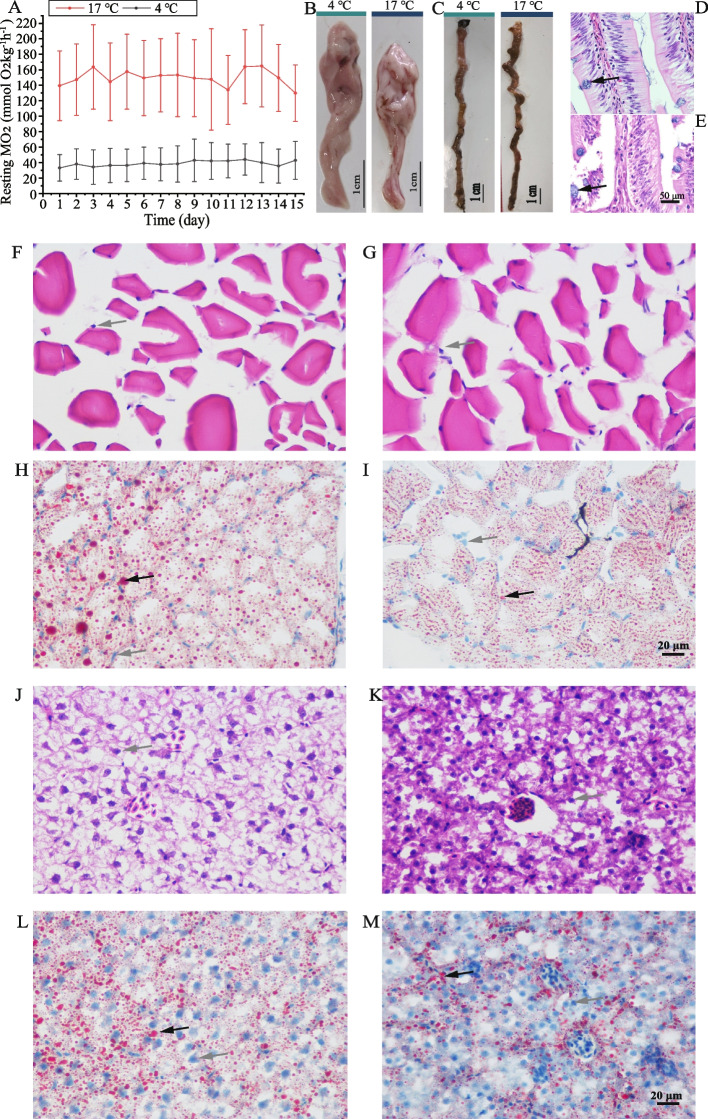
Effects of 15-day cold stress on routine metabolic rate, morphology of hepatopancreas, intestine, and intestine, and histology of hepatopancreas and muscle in *G. przewalskii*. **A** Resting metabolic rates of O_2_ consumption (MO_2_) were decreased by 4 °C environment. Data are shown as means ± SD. **B** & **C** Anatomical morphologies of hepatopancreas and intestine in 4 °C or 17 °C environment after a 15-day acclimation. Scale bar, 1 cm. (D&E) Histomorphology of the intestine stained with H&E at 17 °C (**D**) and 4 °C (**E**). The pinocytotic vesicle with a chyme particle center was labeled by a dark arrow. Scale bar, 50 µm. **F**–**M** Skeletal muscle (**F**–**I**) and hepatopancreas (**J**–**M**) stained with H&E (**F**, **G**, **J**, and **K**) or oil red O (**H**, **I**, **L**, and **M**). The gray arrow marked hyperchromatic cell nucleus and the dark arrow marked oil drops. Scale bar, 20 µm

All fish appeared healthy during the experiment with a 100% survival rate. The average daily feed intake (ADFI) was significantly decreased in CA-treated fish (*P* < 0.01) (0.164 ± 0.075 g/day in CA and 0.552 ± 0.140 g/day in NT), while the frequency of automatic eye reflex significantly increased in CA-treated fish (*P* < 0.01) (5.42 ± 1.17 times/min in CA and 1.88 ± 0.36 times/min in NT). No significant difference was observed in the initial body weight and condition between fish in the CA and NT conditions. CA showed significantly lower FWB, FCF, WG, HIS, and ISI levels (all *P* < 0.01) (Table 1). Blood glucose (Glu) levels significantly increased (*P* < 0.01), while triglyceride (TG) levels decreased in CA-treated fish (*P* < 0.01) (Table 1). The mean MO_2_ (38.82 ± 22.25 mmol O_2_kg^−1^ h^−1^) of fish in CA conditions was obviously (*P* < 0.01) lower than that of fish in NT conditions (149.78 ± 48.98 mmol O_2_kg^−1^ h^−1^) and was retained within a specific range during the period (Fig. 2A).

**Table 1:** Growth indexes and somatic parameters in juvenile *G. przewalskii* exposed at different temperatures for 15 days

Parameters	Temperature		*P-*value
	4 °C	17 °C	
IBW^a^ (g)	6.34 ± 0.87	6.37 ± 0.93	0.791
FBW^b^ (g)**	6.33 ± 0.83	6.80 ± 1.16	< 0.01
ICF^c^ (%)	1.07 ± 0.08	1.10 ± 0.09	0.117
FCF^d^ (%)**	1.15 ± 0.05	1.11 ± 0.08	< 0.01
WG^e^ (%)**	-0.13 ± 0.01	6.26 ± 0.15	< 0.01
HSI^f^ (%)**	7.04 ± 0.25	6.52 ± 0.22	< 0.01
ISI^g^ (%)**	7.28 ± 0.46	6.02 ± 0.13	< 0.01
Glu^h^ (mmol/L)**	10.70 ± 2.09	6.06 ± 1.54	< 0.01
TG^i^ (mmol/L)**	1.36 ± 0.37	2.55 ± 0.51	< 0.01

Values are means of triplicate groups ± SD.  ** means the difference between groups (4 °C vs. 17 °C) is  significant. The asterisks followed by different parameters are significantly different by the Tukey test (* means 0.01 < *P* < 0.05; ** means *P* < 0.01)

^a^IBW = Initial body weight (g)

^b^FBW = Final body weight (g)

^c^ICF = Condition factor

^d^FCF = Condition factor

^e^WG = Percent weight gain (%)

^f^HSI (Hepatosomatic index) = 100 × liver weight/fish weight

^g^ISI (Intestine Somatic Index) = 100 × Intestine weight/fish weight

^h^Glu means blood glucose

^i^TG means triglyceride

### Tissue histology analysis

The anatomical examination revealed that fatty infiltration occurred in the hepatopancreas of NT-treated fish with a pale white surface, while the hepatopancreas of CA-treated fish exhibited a pink appearance and tensile strength (Fig. 2B). Currently, there is no discernible difference in the intestine appearance between CA- and NT-treated fish, except digested chyme in the intestine cavity of fish in NT conditions (Fig. 2C). The intestinal histology of CA-treated fish showed a proportion of small pinocytotic vesicles with a chyme particle centre located in the intestinal villus (Fig. 2D), which was significantly less than that in NT-treated fish (*P* < 0.05) (Fig. 2E). No visible difference was detected in the muscle fibre section between CA- and NT-treated fish (Fig. 2F and G), according to HE staining. The oil drops in the muscle fibre were significantly reduced in CA-treated fish according to the Oil Red O staining (Fig. 2H and I), while NT-treated fish showed large lipid vacuolations with slight hyperaemia swollen hepatocytes (Fig. 2J and L), and several tiny oil droplets were dispersed in hepatocytes, while hepatocytes with moderately depleted lipids were observed in CA-treated fish (Fig. 2K and M).

### Differential gene expression induced by cold stress

A total of 48 RNA libraries (24 from eight tissues of CA-treated fish and 24 from eight tissues of NT-treated fish) were prepared and analysed. A total of 490.66 G raw data were obtained from 48 libraries; the mean GC content was 46.54%, and the mean Q30 was 94.07% (Table S1). The raw reads were deposited in the National Center for Biotechnology Information (NCBI) Sequence Read Archive (SRA) database (will be uploaded). After filtering the low-quality sequences, approximately 56.8 M clean reads were mapped to the reference genome, with a mapping ratio of 77.92–86.30% (Table S2). Approximately 69.87% reads were mapped to the exons of the genome (Figure S1). The sample correlation matrix generated from the gene expression data indicated suitable correlations between the biological replicates (Pearson’s correlation coefficient range was 0.879–0.987; Figure S2). The cluster analysis of samples based on the distance matrix and results of the principal component analysis (PCA) demonstrated a marked discrepancy among the different sample types (Figure S3), suggesting a significant difference in gene expression in different tissues and under various temperature conditions. According to sequence homology analysis, 3,568 new genes of *G. przewalskii* were annotated by BLAST with six public databases (Table S3).

**Fig. 3:**
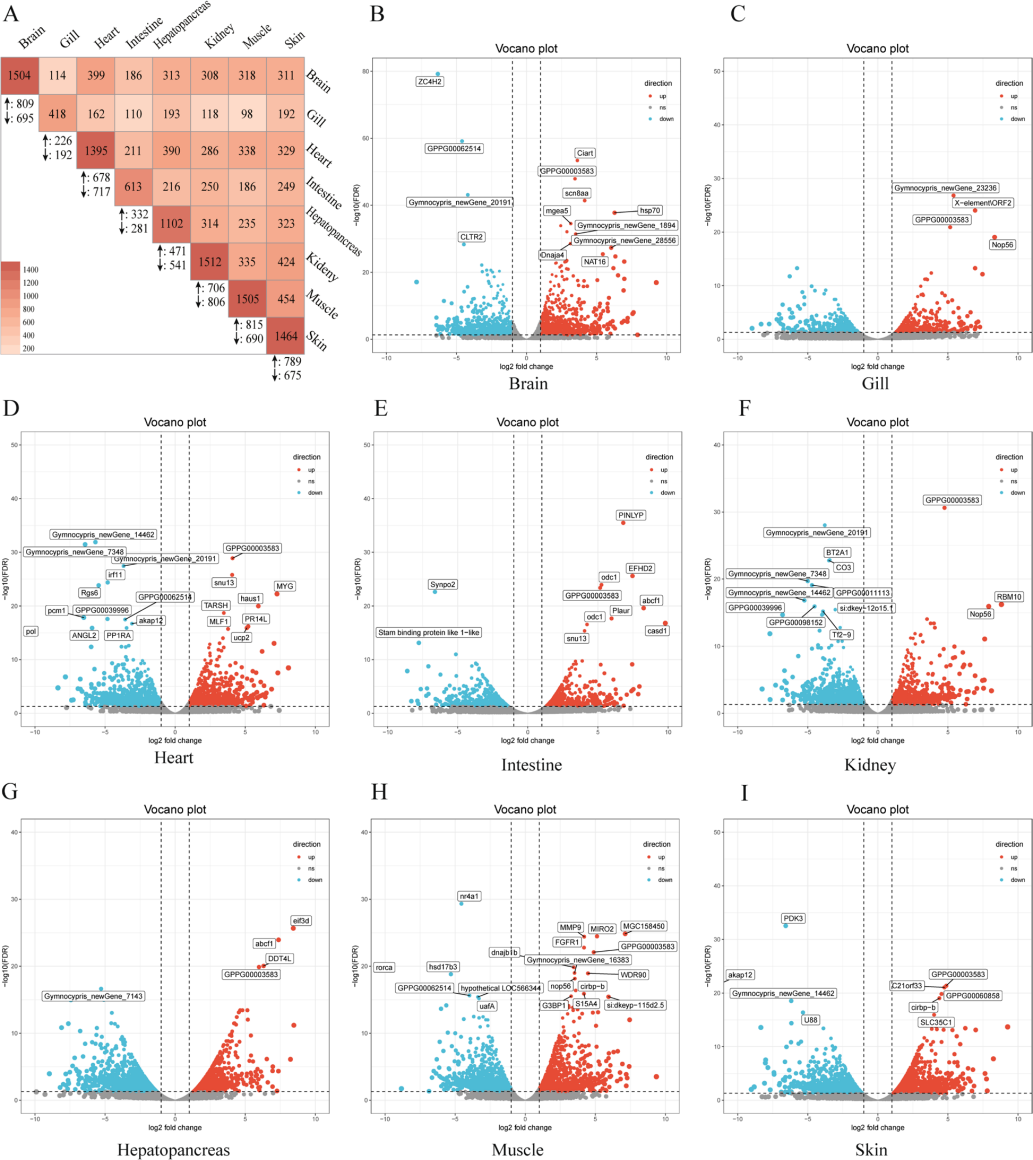
Cold-regulated gene expression in the different tissues of *G. przewalskii*. **A** Matrix heatmap plot indicates the number of DEGs in different tissues, and the common DEGs in any two tissues. **B**–**I** Volcano plots indicate the up- and downregulated genes in the brain (**B**), gill (**C**), heart (**D**), intestine (**E**), kidney (**F**), hepatopancreas (**G**), muscle (**H**), and skin (**I**), respectively. The top up- and downregulated genes are shown in orange and sky-blue, respectively

The FPKM method was applied to calculate the expression profiles. Herein, we detected 5,745 cold-induced DEGs. The DEGs in different tissues and the intersection of any two tissues are displayed in Fig. 3A. Under 15-day cold conditions, the brain (with 1504 DEGs), heart (with 1395 DEGs), kidney (with 1512 DEGs), muscle (with 1505 DEGs), and skin (with 1464 DEGs) demonstrated more DEGs than the gills (with 418 DEGs) and intestine (with 613 DEGs), suggesting a substantial number of transcriptional changes take place in response to cold conditions. The eight tissues shared 25 up- and 10 downregulated genes, representing the typical gene expression changes of different tissues in response to cold stress. Most DEGs were tissue specific, indicating important effects of the tissue type on the responses to cold stress (Figure S4). The DEGs in the eight tissues are displayed in a volcano plot in Fig. 3B–I.

### GO enrichment and KEGG pathway analysis of DEGs

GO and KEGG enrichment analyses were performed to provide insights into the functions of DEGs. The DEGs of the eight tissues mapped to the biological process, cellular component, and molecular function categories are listed in Table S5. The common significantly enriched KEGG pathway in the eight tissues was mapped to alanine, aspartate, and glutamate metabolism (ko00250), spliceosome (ko03040), RNA transport (ko ko03013), phagosome (ko04145), glycolysis/gluconeogenesis (ko00010), and glutathione metabolism (ko00480). Most genes involved in the above pathways were upregulated by cold conditions (Figure S6). Furthermore, the pathways related to carbohydrate metabolism, such as pyruvate metabolism (ko00620) and citrate cycle (TCA cycle) (ko00020), were significantly enriched in the brain. The pathways related to fatty acid metabolism and regulation, such as fatty acid biosynthesis (ko00061), fatty acid degradation (ko00071), fatty acid metabolism (ko01212), peroxisome proliferator-activated receptor (PPAR) signalling pathway (ko03320), and biosynthesis of unsaturated fatty acids (ko01040), were significantly enriched in one or multiple tissues, including heart, hepatopancreas, intestine, kidney, and muscle (Table S6).

### qRT‒PCR validation of DEGs

To verify the reliability of the transcriptome data, we performed qRT‒PCR analysis on 12 cold-induced genes (Figure S6 A). The correlation analysis between RNAseq (log2-fold-change; CA *vs*. NT) and qRT‒PCR (the relative expression ratio; CA *vs*. NT) revealed that most of these genes shared similar expression tendencies with those from the transcriptomic data, with an R^2^ = 0.8533 (Figure S6 B). Hence, the RNA-seq analysis results showed high reproducibility and reliability and would be useful for subsequent studies.

To clarify the time effect during the cold challenge, we identified the change process of relative transcript levels of 12 genes at 4 °C for 0 h, 12 h, 24 h, 48 h, 7 d, 10 d, and 15 d in the hepatopancreas by qRT‒PCR (Figure S7). These genes are involved in the antioxidant process (including glutathione peroxidase *Gpx1a*, oxidation resistance protein 1 *Oxr1*, and superoxide dismutase S*od3a*), glycolysis/gluconeogenesis (including glucose-6-phosphatase 3 *G6pc3*, hexokinase 2 *Hk2*, and phosphofructokinase *Pfpk*), fatty acid oxidation (including long-chain-fatty-acid CoA ligase-1 *Acsl1a*, carnitine palmitoyltransferase-1 *Cpt1a*, and fatty acid binding protein-2 *Fabp2a*). Antioxidant-related genes showed the highest levels of expression at 24 h or 48 h, then tends steadily to low levels after 7 d. The genes involved in glycolysis/gluconeogenesis and fatty acid oxidation showed significant transcriptional expression changes in the first 48 h and then generally levelled off at 10 d and 15 d.

A tendency to rise and then fall.

### Cold-induced differential metabolite analysis

The total ion current (TIC) visual examination of all samples revealed a strong instrumental analysis signal, a large peak capacity, and suitable retention time reproducibility (Figure S8). The composition of essential metabolites in the hepatopancreas, intestine, and muscle in the CA or NT group was determined by LC‒MS, and 583 compounds were identified in the three tissues. For the metabolites, a Pearson’s correlation coefficient matrix of all samples was assessed to determine the correlation between the biological replicates. The results showed that the r^2^ of pairwise comparison within one group was > 0.979, indicating reliable biological repetition (Figure S9). The principal component analysis (PCA) combined with orthogonal projections to partial least squares (OPLS-DA) grouped all samples into distinct clusters, reflecting the obvious differences between the three tissues of fish in the CA and NT groups (Figure S10 and S11).

**Fig. 4:**
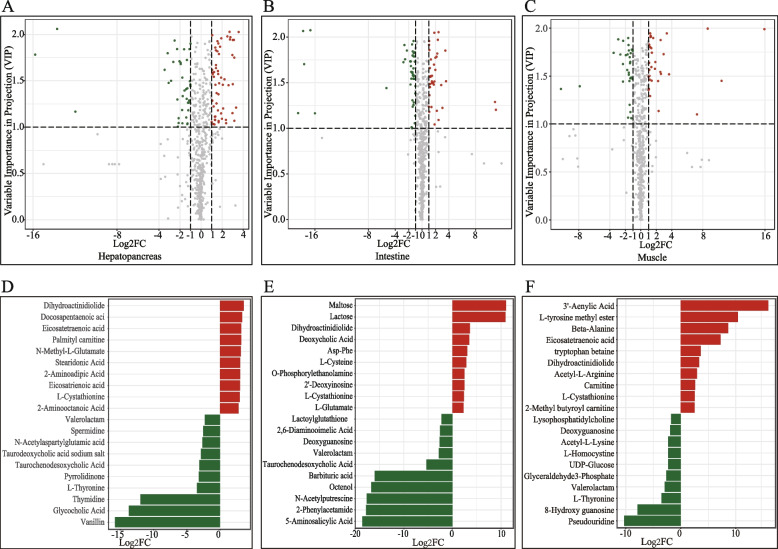
Cold-induced differential metabolites in the hepatopancreas, intestine, and muscle of *G. przewalskii*. The top 10 up- and 10 downregulated metabolites in the hepatopancreas (**A**), intestine (**B**), and muscle (**C**). The upregulated metabolites are represented by red bar and the downregulated metabolites are represented by green bar. Volcano plots indicate the significant up- and down regulated metabolites in the hepatopancreas (**D**), intestine (**E**), and muscle (**F**), respectively. The top up- and downregulated metabolites are shown in orange or green spots, respectively

Combined multidimensional and unidimensional analyses were used to explore the differentially expressed metabolites (DEMs) between the CA and NT groups in the hepatopancreas, intestine, and muscle (Fig. 4A, B and C). A total of 97 DEMs were identified in the hepatopancreas with 58 upregulated metabolites and 39 downregulated metabolites; 82 DEMs were identified in the intestine with 36 upregulated metabolites and 46 downregulated metabolites; and 66 DEMs were identified in muscle with 36 upregulated metabolites and 30 downregulated metabolites. The top 10 upregulated metabolites and 10 downregulated metabolites of the hepatopancreas, intestine, and muscle are listed in Fig. 4D, E and F, respectively. A total of 14 common metabolites were shared in the three tissues, including 4-hydroxy-L-glutamic acid, glutamate-cysteine, deoxyguanosine, GDP-L-fucose, L-cystathionine, 5-methylcytosine, 2-aminooctanoic acid, N6-succinyl adenosine, 2-aminoadipic acid, palmitoylcarnitine, valerolactam, dihydroactinidiolide, and N-methyl-L-glutamate. Most of these are classified into amino acids, nucleotides, and their derived metabolites.

### Integrated analysis of the transcriptome and metabolome

**Fig. 5:**
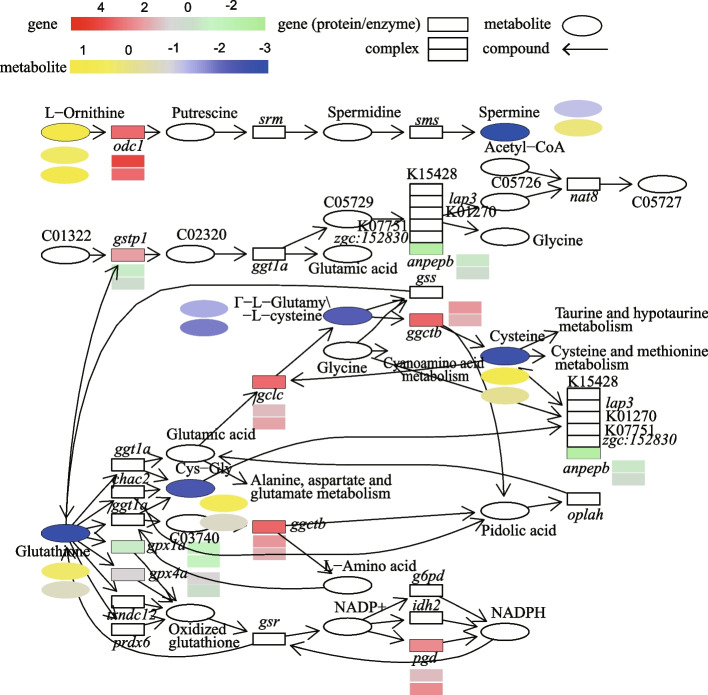
Glutathione metabolism pathway with the mapped DEGs and DEMs. The glutathione metabolism-related DEGs and DEMs in the hepatopancreas, intestine, and muscle are mapped into the glutathione metabolism pathway. DEGs and DEMs in hepatopancreas are located in the squares (genes) or ellipses (metabolites) on the pathway. DEGs and DEMs in intestine (upper) and muscle (below) are located beside the symbols of genes or metabolites. The symbols of DEGs are denoted by italicized texts, and DEMs are denoted by roman numerals

A total of 612, 1101, and 1504 DEGs and 82, 97, and 66 DEMs in the intestine, hepatopancreas, and muscle, respectively, were used to analyse the correlations between genes and metabolites. A total of 359 DEGs and 78 DEMs in the intestine, 942 DEGs and 87 DEMs in the hepatopancreas, and 855 DEGs and 61 DEMs in muscle showed high correlations (Pearson’s correlation coefficient > 0.8) (Figure S12, S13 and S14). The common KEGG pathways enriched by both DEGs and DEMs in the hepatopancreas, intestine, and muscle were obtained. Glutathione metabolism (ko00480) was shared by the abovementioned tissues (Fig. 5), arginine and proline metabolism (ko00330) was shared by the intestine and muscle, and ascorbate and aldarate metabolism (ko00053) was shared by the hepatopancreas and muscle. Fructose and mannose metabolism (ko00051), primary bile acid biosynthesis (ko00120), purine metabolism (ko00230), amino sugar and nucleotide sugar metabolism (ko00520), and glycerophospholipid metabolism (ko00564) were enriched in the intestine. Alanine, aspartate, and glutamate metabolism (ko00250), lysine degradation (ko00310), histidine metabolism (ko00340), tryptophan metabolism (ko00380), beta-alanine metabolism (ko00410), glyoxylate and dicarboxylate metabolism (ko00630), aminoacyl-tRNA biosynthesis (ko00970), carbon metabolism (ko01200), PPAR signalling pathway (ko03320), circadian rhythm (ko04710), insulin signalling pathway (ko04910), GnRH signalling pathway (ko04912), and pancreatic secretion (ko04972) were enriched in the hepatopancreas, and the DEGs and DEM mapped pathways were upregulated. Valine, leucine, and isoleucine degradation (ko00280) and mineral absorption (ko04978) were enriched in the muscle. DEGs and DEMs enriched in these KEGG pathways are shown in Figure S15.

## Discussion

Herein, we provided a comprehensive analysis of the molecular network underlying the low-temperature adaptation of *G. przewalskii* by integrating metabolomic and transcriptomic data and combining behavioural, physiological, and histological results. These findings provide comprehensive insights into the molecular mechanism of the physiological metabolism changes that facilitate the excellent low-temperature adaptation of Tibetan Plateau fish.

Since most fish are ectotherms, their physiology is strongly affected by temperature with respect to the metabolic rate and thus the energy balance and behaviour, including locomotor and feeding behaviour. In this study, we observed that food intake and RMR decreased in the CA group, while swimming ability and evasive capture behaviour were not significantly different between the CA and NT groups. In addition, fewer food vacuoles were detected between intestinal villi in the CA group than in the NT group, proving that 4 °C cold stress suppresses digestion and absorption within the gastrointestinal tract [57]. Interestingly, body weight was not significantly different in the CA group, and glucose was maintained at an unexpectedly higher level than that in NT group. This finding was opposite to that previously reported in *Chanos chanos* [58], *Piaractus mesopotamicus* [59], *E. coioides* [23], and *Oreochromis niloticus* [60], wherein the blood glucose levels significantly decreased when the fish were exposed to chronic low temperature for 2 (48 h) to 21 days. Our results indicated a potential specific cold physiological response mechanism in *G. przewalskii*. Thus, high blood glucose could be a critical physiological characteristic of *G. przewalskii* for survival under cold conditions. The oil droplet losses in hepatocytes combined with decreased TG levels demonstrated cold stress-induced lipid metabolism. Similar results were observed in *Takifugu obscurus* [24] and *E. coioides* [23]. Adjusting behaviour and physiology, such as reducing RMR and feeding and activating fat mobilization, allowed *G. przewalskii* to adequately adapt to a low-temperature environment through physiological homeostasis remodelling.

The species-specific and tissue-specific expression patterns of cold-induced DEGs have been demonstrated in several teleosts, such as *D. rerio* [34], *C. carpio* [25], and *O. niloticus* [61]. We also found abundant tissue-specific DEGs in *G. przewalskii*, indicating a complex and diverse functional collaboration modification when the fish are exposed to cold stress. A total of 25 upregulated and 10 downregulated DEGs were shared by the eight studied tissues, indicating a nontissue-specific response to cold stress in *G. przewalskii*. Among these, the RNA binding motif protein 8A gene (*rbm8a*), PRP31 pre-mRNA processing Factor 31 homologue gene (*prpf31*), and high-mobility group Box 3a gene (*hmgb3a*) were also identified in *D. rerio* [24, 37, 62] and *C. carpio* [25], indicating the presence of core cold stress-response genes in fish. In addition, the cold-inducible RNA binding protein gene (*cirbpa/b*), heat shock cognate 70-kd protein gene (*hsp70*), eukaryotic translation initiation factor gene (*eif*), ATP-binding cassette transporters gene (*abcf1*), and U4/U6snRNA coding gene (*snu13*) were highly expressed in one or more tissues of the brain, heart, intestine, hepatopancreas, muscle, and skin. These genes have been proven to have a biomarker response to cold stress in fish [62, 63]. However, it should be noted that the expression changes of genes such as *hsp70*, *abcf1*, *snu13*, and *eif* could also be induced by other stress responses, including high temperature [63], inflammation [64], hypoxia [65] and oxidative stress [66].

Functional categories including spliceosomes and proteasomes were highly enriched in the DEGs of the eight tissues. Alternative splicing (AS) is an essential posttranscriptional regulatory mechanism of cells that generates transcript variability and proteome diversity [61]. In *G. przewalskii*, KEGG analysis found that the spliceosome was a highly enriched pathway in the DEGs that was upregulated under cold stress. Long et al. [62] demonstrated that upregulated genes are highly enriched in the spliceosome under cold stress in *D. rerio*. These results demonstrated that activated AS might be a critical and universal biological process used by fish to cope with cold stress. Many RNA-binding proteins (RBPs), such as the serine/arginine-rich (SR) and heterogeneous nuclear RNP (hnrnp) protein families [67], are critical for AS regulation. In this study, six SR genes (*srsf1a*, *srsf1b*, *srsf2*, *srsf5a*, *srsf5b*, and *srsf5c*), one RNP gene (*hnrnp*), and two RBP genes (*cirbpa* and *cirbpb*) were regulated by cold stress. These findings suggested that RBPs act in AS-related stress regulation in fish.

LC‒MS metabolomics and transcriptomics were integrated to investigate the cold-triggered metabolic regulatory changes in the hepatopancreas, intestine, and muscle. Additional DEMs were detected in the hepatopancreas (97 DEMs) and intestine (82 DEMs) compared with muscle (66 DEMs), indicating that the active metabolites in digestive organs/tissues, hepatopancreas, and intestine display a critical functional response to cold stress. The liver (named hepatopancreas in cyprinids) is the central organ involved in energy homeostasis in the body [68]. In *E. coioides*, cold stress increased liver lipid metabolism and induced the expression of genes related to lipid metabolism [23]. In *N. albiflora*, the enzymes involved in carbohydrate metabolism, lipid metabolism, and the digestive system were enriched to resist low temperatures [69]. In this study, we also found that several substrates and derivatives for energy metabolism were upregulated in the hepatopancreas, including carbohydrate metabolites (such as hydroxyphenyl lactic acid) and amino acid metabolites (such as 4-hydroxy-L-glutamic acid and α-ketoglutaric acid). Nucleotide metabolites (such as 1-methylxanthine and 2’-deoxyinosine), organic acids and their derivatives (such as ethylmalonate and 4-acetamidobutyric acid), and several long-chain fatty acids (C > 20) (such as behenate and lignocerate) indicated that *G. przewalskii* might adapt to cold stress by altering energy metabolism. *D. rerio* might synthesize abundant C24:0 via *elovl1* to promote mitochondrial *β*-oxidation and provide the required energy for resisting the cold environment [70]. In addition, lignocerate (C24:0) and the key gene *elovl1a/b* increased in the hepatopancreas, intestine, and muscle. Therefore, we inferred that C24:0 might be a major energy source for *G. przewalskii* while adapting to cold stress. The desaturation of fatty acids is a crucial adaptation mechanism for fish to maintain membrane fluidity under cold stress. Additionally, an increase in the poly unsaturated fatty acid (PUFA) level in several tissues has been observed in many fish, including *C. carpio* [71] and *C. chanos* [72, 73], under cold conditions. In this study, α-linolenate (18C:3N3) and arachidonate (RAR, 20C:4n6) were upregulated in CA-treated fish, indicating a cellular structure change under cold stress. Docosahexaenoate (DHA) plays a key role in the structure of cell membranes, ion balance, regulation of reproduction, and the function of the immune system [74], which is essential for normal growth, health, and development in all vertebrates, including fish [75]. Moreover, DHA was highly abundant without a significant difference between the CA and NT groups, suggesting that stable DHA homeostasis under cold conditions might be critical for the survival of *G. przewalskii*.

Cold stress-induced oxidative stress or oxidative damage has been proven in *T. obscurus* [24], *Sparus aurata* [76], and *Gasterosteus aculeatus* [77]. High oxidative stress may originate from two major factors: high dissolved oxygen (OD) levels since a physical trait of cold aquatic environments is having high saturation levels of dissolved oxygen in well-mixed surface waters [78] and the accumulation of reactive oxygen species [79, 80]. The glutathione antioxidant system is the primary enzymatic antioxidant defence system of living organisms. It reduces hydrogen peroxide and lipid hydroperoxides at the expense of oxidizing reduced glutathione (GSH) to oxidized glutathione (GSSG) by glutathione peroxidase (GPx) [81]. The synthesis of GSH depends on the cellular concentrations of glutamate and glutamine [82]. Glutathione metabolism alterations are shown in discus fish gills [40], gilthead seabream liver [39], and pufferfish liver [42] under cold stress, as revealed by metabolomics. In the present study, cold-induced GSH and several key genes involved in the glutathione metabolism pathway were detected in the intestine and muscle of *G. przewalskii*, indicating that the glutathione antioxidant system was triggered by cold stress to resist oxidative damage. We also found that the levels of several sulfur-containing amino acids and related derivatives, including cysteine (Cys), cysteinyl glycine (Cys-Gly), glutamylcysteine (Γ-L-Glutamy-L-cysteine), glutamate-cysteine (Glu-Cys) and L-cystathionine, significantly changed under cold stress. These amino acids could provide sulfhydryl (-SH), which is involved in glutathione synthesis and antioxidant processes. In addition, there was no significant difference observed in GSSG levels between the CA and NT groups, while the phosphogluconate dehydrogenase gene (*pgd*) was significantly upregulated in the CA group. In the pentose phosphate pathway (PPP), GPD catalyses the conversion of glucose-6-phosphatase to gluconate-6P, and this step is accompanied by the production of NADPH [83]. NADPH is the critical reducing power that can reduce GSSG to GSH via oxidation [84, 85]. Therefore, we inferred that *pgd* sustained NADPH (no significant difference between the CA and NT groups) that catalysed GSSG into GSH. Cold stress could induce protective responses through the GSH antioxidant system in fish [43], and the dynamic change in GSH-GSSG provided a robust antioxidant capacity in maintaining the redox balance [40]. Overall, these differential metabolites reflect the complex transport compensation mechanism of *G. przewalskii* in response to cold stress.

Fatty acid anabolism and catabolism, including fatty acid elongation, fatty acid degradation, biosynthesis of unsaturated fatty acids, and *β*-oxidation of fatty acids, were activated in multiple tissues, indicating a nontissue-specific fatty acid-dependent energy utilized in cold stress. Mitochondrial *β*-oxidation is activated at low temperatures to provide the required energy for resisting the cold environment, which has also been confirmed in many fish species [60, 71, 86]. Combined with the tissue analyses, we hypothesize that the decrease in lipid droplets could be associated with increased lipolysis of the higher molecular weight lipids, resulting in increased levels of fatty acids [87]. Then, SFAs and MUFAs could be used for oxidative metabolism to produce energy [71, 88], and PUFAs could be used as a component of biological membranes [71–73]. We also found several cold-induced DEGs involved in fatty acid elongation and biosynthesis. Similar findings were recorded in *C. carpio* [87], *Misgurnus anguillicaudatus* [89], and *E. coioides* [90]. Therefore, we inferred that fatty acid elongation and biosynthesis could also be associated with the accumulation of fatty acids in response to cold stress. We found that different tissues show diverse energy usage preferences. The glycolysis/gluconeogenesis pathway and pyruvate and lactic acid metabolism were activated in the brain, indicating a carbohydrate metabolism preference under cold conditions. Although fatty acid metabolism provides primary energy for the physiological function of most tissues, information about lipids and amino acids as energy sources in the fish brain is scarce. Free fatty acids do not have free access to the brain since the blood‒brain barrier isolates the brain from systemic circulation [91], while glucose and other monocarboxylates can be transported into the brain and generate ATP through a series of successive oxidative phosphorylation processes [92]. Environmental factors induce changes in brain energy parameters in teleosts; for example, cold stress alters substrate utilization [91]. Lactate utilization and transport pathways are crucial to maintain the vital functions of the brain during cold stress in *D. rerio* [93]. Therefore, we inferred that the elevated blood glucose levels with active glycolysis, pyruvate, and lactic acid metabolism are critical for energy homeostatic features in the brain to ensure the optimal function of the central nervous system with active behaviour and suitable coordination of *G. przewalskii* under cold conditions. In the muscle, the levels of isoleucine and valine increased under cold stress. Branched-chain amino acids (BCAAs), leucine, isoleucine, and valine, are involved in fish physiology [94]. Isoleucine and valine regulate protein synthesis and degradation and have a preponderant role in energy homeostasis [95]. The cold-induced elevated isoleucine and valine could be involved in cold metabolic adaptation in muscles.

The functions of specific tissues were also adjusted to adapt to cold stress. The cardiac muscle contraction pathway was significantly enriched in the heart. Chronic (days to weeks) cold exposure triggers temperature-dependent remodelling in the fish heart to preserve the active pumping properties [96]. Cold acclimation could alter the gene transcript levels of cardiac myofilament proteins in the trout heart that control contractile function [97]. Such a strategy may contribute to maintaining contractile function in the trout heart during cold acclimation [97]. In the pearl gentian grouper, cardiac muscle contraction was detected based on RNA-seq results and could be physiologically adjusted to reduce damage and maintain essential life functions under cold stress [98]. Herein, we found that the cardiac muscle contraction pathway was significantly enriched in the heart DEGs and that key genes, including cytochrome c oxidase subunit 7A2 gene (*Cox7a2*) and cytochrome c oxidase subunit 5A gene (*Cox5a*), were upregulated by cold stress. These results indicated that functional remodelling regulated by multiple genes, which might be triggered in the heart of *G. przewalskii,* may be an essential cold adaptation to maintain blood circulation.

Both DEGs and DEMs were significantly enriched the PPAR pathway in the hepatopancreas. In the liver of *N. albiflora*, *L. crocea*, and carp species, the expression of genes related to the PPAR signalling pathway, including *PPARα*, *SCD-1*, *LXRα*, *FATP1/4*, *LPL*, *CPT-1*, *ACO*, *Perilipin*, and *CAP*, was upregulated during cold stress [36, 69, 99]. PPARs are ligand-activated transcription factors that belong to the nuclear receptor superfamily and regulate the expression of target genes involved in lipid and energy metabolism [91, 100, 101]. In addition, PPAR family members showed diverse expression patterns under cold conditions. Among them, *PPARγ* was upregulated under cold conditions, consistent with its target genes, including *cpt1a*, *acsbg2*, pck1, and *acsl1a*, while *PPARα* was downregulated by cold stress. Moreover, the ligand compounds of PPARs also showed variable changes. These results indicated a functional differentiation of *PPARs* and their ligands, requiring in-depth investigation.

In this study, we used a cold acclimation period of 15 days to detect transcriptomic and metabolomic changes induced by a cold environment. The same experimental period or a similar duration, such as two weeks, is often selected [102] because it has been reported that a rapid establishment of the new steady state from previous homeostasis in critical physiological and biochemical processes requires approximately two weeks in fish after low temperature stress [103–105]. The expression patterns of genes involved in stress response, fatty acid metabolism, glycometabolism, and antioxidant activity by qRT‒PCR in this study indicated that an establishment of new homeostasis at the transcriptional level occurred in the ten days after the cold challenge. In addition, we selected 17 °C as a controlled temperature because this temperature (or another similar temperature such as 16 °C [106]) is commonly considered an optimal temperature for the survival and growth of *G. przewalskii* [106–108]. Under natural ecological conditions, 17 °C is also an expected temperature in the growing season of *G. przewalskii* [5]. Therefore, this temperature (or a similar temperature) is always applied to large-scale artificial conservation and biological studies of *G. przewalskii* and routine temperature treatment [106–108]. However, a long-term feeding condition at 17 °C or a similar temperature could limit the fish’s health potential due to substantial fat deposition in the hepatopancreas. Whether an accumulation of energy due to permanent breeding at an optimum temperature is detrimental to health needs further study.

## Conclusion

This study suggested that cold stress resulted in significant changes in the gene expression and metabolic profiles of multiple tissues in *G. przewalskii*. The findings elucidated the behavioural and physiological responses involved in the cold adaptation of Tibetan Plateau fish. Blood Glu levels increased, while blood TG levels significantly decreased under cold conditions. Alanine, aspartate, and glutamate metabolism (ko00250), spliceosome (ko03040), RNA transport (ko03013), phagosome (ko04145), glycolysis/gluconeogenesis (ko00010), and glutathione metabolism (ko00480) were significantly enriched in the DEGs of the eight tissues. A GSH-GSSG-dependent antioxidant biological process was triggered to resist peroxide accumulation under cold conditions. Fatty acid anabolism and catabolism, including fatty acid elongation and degradation and biosynthesis of unsaturated fatty acids, were activated in multiple tissues, indicating nontissue-specific FA-dependent energy utilization during cold stress. Carbohydrate metabolism is critical for homeostatic energy features in the brain, BCAA metabolism is crucial for energy regulation in muscle, and myocardial function remodelling is essential to the circulatory system. These phenomena indicated a multitissue collaboration network regulated by multiple genes contributing to cold adaptation in *G. przewalskii*. In this study, we investigated the tissue-specific changes in the transcriptome and metabolome under cold stress. However, plasma could maintain homeostatic concentrations, blood indicators and metabolites could reflect the whole state and the antioxidant system exists in circulation and plays crucial roles in the response to oxidative stress, which deserves further study. In addition, we investigated changes in physiological, metabolomic and transcriptomic metabolic pathways induced by artificial cold challenge simulating the ecological environment. However, the physiological responses of wild fish to low-temperature stress are influenced by age, sex, physiological status, nutrition, and thermal memory. Therefore, our results could partially reflect the changes in the natural ecological environment.

### Supplementary Information

**Additional file 1: Table S1.** Sequencing data statistics. **Table S2.** Mapped clean reads statistics. **Table S3.** Statistics of novo genes annotation. **Table S4.** Primers used to qRT-PCR.

**Additional file 2.**

**Additional file 3.**

**Additional file 4.**

**Additional file 5.**

**Additional file 6.**

**Additional file 7.**

**Additional file 8.**

**Additional file 9.**

**Additional file 10.**

**Additional file 11.**

**Additional file 12.**

**Additional file 13.**

**Additional file 14.**

**Additional file 15.**

**Additional file 16.**

**Additional file 17.**

**Additional file 18.**

**Additional file 19.**

## Data Availability

Reads of resequencing data were deposited in NCBI database under project number PRJNA843344.
