# Supplementary material for: Physiological, metabolomic, and transcriptomic reveal metabolic pathway alterations in Gymnocypris przewalskii due to cold exposure
Source: BMC Genomics. 2023 Sep 14;24:545. doi: 10.1186/s12864-023-09587-9 (PMC10500822; doi:10.1186/s12864-023-09587-9)
Supplement: Supplementary file 1 — Additional file 1: Table S1. Sequencing data statistics. Table S2. Mapped clean reads statistics. Table S3. Statistics of novo genes annotation. Table S4. Primers used to qRT-PCR. [file 12864_2023_9587_MOESM1_ESM.docx]

**Table S1 Sequencing data statistics**

| **Samples** | **Read pair Number** | **Base Number** | **GC Content** | **%≥Q30** |
| --- | --- | --- | --- | --- |
| LB1 | 34,029,755 | 10,208,926,500 | 45.61% | 94.07% |
| LB2 | 38,807,698 | 11,642,309,400 | 45.75% | 95.02% |
| LB3 | 35,314,012 | 10,594,203,600 | 45.77% | 94.55% |
| LG1 | 29,242,400 | 8,772,720,000 | 46.10% | 93.73% |
| LG2 | 30,873,440 | 9,262,032,000 | 46.50% | 94.32% |
| LG3 | 30,025,590 | 9,007,677,000 | 46.35% | 94.01% |
| LH1 | 26,362,436 | 7,908,730,800 | 46.18% | 94.02% |
| LH2 | 30,713,450 | 9,214,035,000 | 46.26% | 94.57% |
| LH3 | 31,976,450 | 9,592,935,000 | 46.15% | 93.80% |
| LI1 | 38,352,928 | 11,505,878,400 | 46.78% | 93.67% |
| LI2 | 38,961,457 | 11,688,437,100 | 47.74% | 94.35% |
| LI6 | 33,075,536 | 9,922,660,800 | 46.70% | 94.21% |
| LK1 | 34,169,644 | 10,250,893,200 | 46.66% | 93.88% |
| LK2 | 49,824,822 | 14,947,446,600 | 46.17% | 94.35% |
| LK3 | 32,927,197 | 9,878,159,100 | 46.17% | 93.92% |
| LL1 | 47,387,147 | 14,216,144,100 | 46.66% | 94.65% |
| LL2 | 34,763,387 | 10,429,016,100 | 46.73% | 94.18% |
| LL3 | 25,841,957 | 7,752,587,100 | 46.90% | 95.07% |
| LM1 | 32,230,446 | 9,669,133,800 | 48.60% | 94.43% |
| LM2 | 32,415,222 | 9,724,566,600 | 48.64% | 93.87% |
| LM3 | 36,069,545 | 10,820,863,500 | 48.24% | 94.04% |
| LS1 | 34,749,292 | 10,424,787,600 | 46.65% | 94.06% |
| LS2 | 30,033,556 | 9,010,066,800 | 46.99% | 94.25% |
| LS3 | 29,779,112 | 8,933,733,600 | 46.89% | 94.14% |
| NB1 | 33,000,944 | 9,900,283,200 | 45.55% | 93.91% |
| NB2 | 30,166,969 | 9,050,090,700 | 45.68% | 93.58% |
| NB3 | 30,568,108 | 9,170,432,400 | 45.25% | 93.74% |
| NG1 | 39,646,707 | 11,894,012,100 | 46.36% | 94.24% |
| NG2 | 32,332,594 | 9,699,778,200 | 46.09% | 93.76% |
| NG3 | 32,850,901 | 9,855,270,300 | 45.73% | 93.99% |
| NH1 | 29,894,672 | 8,968,401,600 | 46.03% | 93.10% |
| NH2 | 32,743,989 | 9,823,196,700 | 46.07% | 94.31% |
| NH3 | 34,798,290 | 10,439,487,000 | 46.35% | 93.65% |
| NI1 | 37,272,900 | 11,181,870,000 | 46.32% | 93.92% |
| NI2 | 37,123,250 | 11,136,975,000 | 46.53% | 93.79% |
| NI3 | 31,013,281 | 9,303,984,300 | 46.72% | 93.86% |
| NK1 | 32,283,180 | 9,684,954,000 | 46.09% | 93.16% |
| NK2 | 34,128,918 | 10,238,675,400 | 45.93% | 93.96% |
| NK3 | 40,242,087 | 12,072,626,100 | 45.94% | 94.08% |
| NL1 | 39,537,612 | 11,861,283,600 | 45.88% | 94.12% |
| NL2 | 39,443,609 | 11,833,082,700 | 46.25% | 93.67% |
| NL3 | 32,988,749 | 9,896,624,700 | 45.40% | 94.09% |
| NM1 | 35,145,014 | 10,543,504,200 | 48.34% | 94.79% |
| NM2 | 31,319,060 | 9,395,718,000 | 47.97% | 94.11% |
| NM3 | 31,295,520 | 9,388,656,000 | 48.06% | 94.35% |
| NS1 | 27,568,750 | 8,270,625,000 | 46.82% | 94.05% |
| NS2 | 35,399,477 | 10,619,843,100 | 46.28% | 93.92% |
| NS3 | 36,830,850 | 11,049,255,000 | 46.87% | 93.99% |

**Table S2 Mapped clean reads statistics**

| **Samples** | **Total Reads** | **Mapped Reads** | **Mapped Ratio** | **Uniq Mapped Reads** | **Uniq Mapped Ratio** |
| --- | --- | --- | --- | --- | --- |
| LB1 | 68,059,510 | 56,420,984 | 82.90% | 9,760,830 | 17.3% |
| LB2 | 77,615,396 | 64,596,886 | 83.23% | 10,658,486 | 16.5% |
| LB3 | 70,628,024 | 59,271,518 | 83.92% | 10,135,430 | 17.1% |
| LG1 | 58,484,800 | 48,706,830 | 83.28% | 7,695,679 | 15.8% |
| LG2 | 61,746,880 | 52,358,346 | 84.80% | 9,110,352 | 17.4% |
| LG3 | 60,051,180 | 50,752,132 | 84.51% | 8,983,127 | 17.7% |
| LH1 | 52,724,872 | 43,097,828 | 81.74% | 7,843,805 | 18.2% |
| LH2 | 61,426,900 | 50,472,046 | 82.17% | 9,286,856 | 18.4% |
| LH3 | 63,952,900 | 51,795,098 | 80.99% | 9,064,142 | 17.5% |
| LI1 | 76,705,856 | 64,541,706 | 84.14% | 11,036,632 | 17.1% |
| LI2 | 77,922,914 | 67,013,064 | 86.00% | 10,454,038 | 15.6% |
| LI6 | 66,151,072 | 55,943,148 | 84.57% | 9,566,278 | 17.1% |
| LK1 | 68,339,288 | 56,434,622 | 82.58% | 9,424,582 | 16.7% |
| LK2 | 99,649,644 | 80,647,248 | 80.93% | 13,306,796 | 16.5% |
| LK3 | 65,854,394 | 53,274,192 | 80.90% | 9,269,709 | 17.4% |
| LL1 | 94,774,294 | 81,072,846 | 85.54% | 14,430,967 | 17.8% |
| LL2 | 69,526,774 | 60,001,276 | 86.30% | 10,860,231 | 18.1% |
| LL3 | 51,683,914 | 44,058,128 | 85.25% | 8,106,696 | 18.4% |
| LM1 | 64,460,892 | 55,250,020 | 85.71% | 9,503,003 | 17.2% |
| LM2 | 64,830,444 | 55,885,524 | 86.20% | 9,109,340 | 16.3% |
| LM3 | 72,139,090 | 60,972,868 | 84.52% | 10,792,198 | 17.7% |
| LS1 | 69,498,584 | 55,929,732 | 80.48% | 9,340,265 | 16.7% |
| LS2 | 60,067,112 | 48,162,510 | 80.18% | 7,946,814 | 16.5% |
| LS3 | 59,558,224 | 48,036,054 | 80.65% | 8,310,237 | 17.3% |
| NB1 | 66,001,888 | 55,390,258 | 83.92% | 9,305,563 | 16.8% |
| NB2 | 60,333,938 | 50,544,772 | 83.78% | 8,542,066 | 16.9% |
| NB3 | 61,136,216 | 51,506,706 | 84.25% | 7,983,539 | 15.5% |
| NG1 | 79,293,414 | 65,734,438 | 82.90% | 11,372,058 | 17.3% |
| NG2 | 64,665,188 | 54,324,994 | 84.01% | 9,615,524 | 17.7% |
| NG3 | 65,701,802 | 55,139,340 | 83.92% | 9,925,081 | 18.0% |
| NH1 | 59,789,344 | 49,068,172 | 82.07% | 8,930,407 | 18.2% |
| NH2 | 65,487,978 | 54,284,022 | 82.89% | 9,879,692 | 18.2% |
| NH3 | 69,596,580 | 57,567,926 | 82.72% | 10,016,819 | 17.4% |
| NI1 | 74,545,800 | 62,272,296 | 83.54% | 10,150,384 | 16.3% |
| NI2 | 74,246,500 | 62,267,186 | 83.87% | 11,021,292 | 17.7% |
| NI3 | 62,026,562 | 51,750,278 | 83.43% | 8,952,798 | 17.3% |
| NK1 | 64,566,360 | 53,470,012 | 82.81% | 9,517,662 | 17.8% |
| NK2 | 68,257,836 | 55,922,102 | 81.93% | 9,842,290 | 17.6% |
| NK3 | 80,484,174 | 66,354,294 | 82.44% | 10,483,978 | 15.8% |
| NL1 | 79,075,224 | 67,504,828 | 85.37% | 11,070,792 | 16.4% |
| NL2 | 78,887,218 | 67,476,902 | 85.54% | 10,863,781 | 16.1% |
| NL3 | 65,977,498 | 55,708,416 | 84.44% | 9,637,556 | 17.3% |
| NM1 | 70,290,028 | 59,771,848 | 85.04% | 10,579,617 | 17.7% |
| NM2 | 62,638,120 | 53,113,186 | 84.79% | 8,976,128 | 16.9% |
| NM3 | 62,591,040 | 52,512,318 | 83.90% | 9,084,631 | 17.3% |
| NS1 | 55,137,500 | 44,359,352 | 80.45% | 8,073,402 | 18.2% |
| NS2 | 70,798,954 | 55,163,434 | 77.92% | 9,929,418 | 18.0% |
| NS3 | 73,661,700 | 58,411,666 | 79.30% | 10,630,923 | 18.2% |

**Table S3 Statistics of novo genes annotation**

| **Annotated databases** | **New Gene Number** |
| --- | --- |
| COG | 1,215 |
| GO | 3,091 |
| KEGG | 1,541 |
| KOG | 3,386 |
| Swiss-Prot | 2,989 |
| nr | 3,560 |
| All | 3,568 |

**Table S4 Primers used to qRT-PCR**

| Gene name | Gene ID | Primers | Product length |
| --- | --- | --- | --- |
| *Elovl1a* | GPPG00000681 | ATCACTCTGTCTTACCCTGGACCTG | 134 bp |
|  |  | TGAAGCAGCCAGACCGTAGTAGG |  |
| *Cpt1a-1* | GPPG00070304 | ATCCCCATGTGCTCATCTCA | 160 bp |
|  |  | GCAGCCGTCCGTCATAAAAC |  |
| *Acsla1a-1* | GPPG00027961 | AAGGACACTCGAAGGACGGA | 164 bp |
|  |  | AGCAGGCCAGCTCTGAAATC |  |
| *Cirbpa* | GPPG00012864 | TGGTGGTGGTGGTGGTGGATAC | 147 bp |
|  |  | TCGTGTGAAGCATAACTGTCGTAGC |  |
| *Scd1* | GPPG00029197 | GCCTTTGGAGCCACAGGTCTTAC | 125 bp |
|  |  | CTTGTGATGGACACGATGGTCTCTG |  |
| *Hspa4* | GPPG00017317 | AGAAACCCGTCGCTGATTGTGTC | 115 bp |
|  |  | CCGTATGCCAGAGCCAAGTTGAG |  |
| *Pparγ-1* | GPPG00012118 | AGTCAGCGAGGTCACCGAGTTC | 149 bp |
|  |  | AGATGAGCGTGCCGTCTTTGTTC |  |
| *Fabp2a* | GPPG00006966 | AACTTTACTCTGGGCGTCACCTTTG | 113 bp |
|  |  | TTGTCCTTGCGTGCGAATGTCC |  |
| *Hmgb3b* | GPPG00054508 | ACAGCCTCACAGATTCCACCAAAC | 119 bp |
|  |  | CTTCGTCTTGTAGTCCGCAGCATC |  |
| *DDX23* | GPPG00055821 | GCAAGAGGAACTGAGGCTGAAGAAG | 149 bp |
|  |  | CTCCTTTGGTCGTGATGCTGTAGTC |  |
| *map3k1* | GPPG00017558 | ATACCAAAGCGAAGCAGCCATACC | 125 bp |
|  |  | GACAGCCATCAGAGTGCCAGTG |  |
| *Pprc1* | GPPG00013157 | CATTGGACCGTGGAAGGCTGAAG | 127 bp |
|  |  | GAACGAGACCTGGAACGAGAAGATG |  |
| *β-actin* | GPPG00046178 | AGGTCATCACCATCGGCAAT | 150 bp |
|  |  | TGGCATACAGGTCCTTACGG |  |
| *Ef-1α* | GPPG00085918 | GCTGCGTTCTGCTCTCGGTAAG | 145 bp |
|  |  | GATGTCGTCCTCTTCCTCCTCCTG |  |
