# Supplementary material for: Physiological, metabolomic, and transcriptomic reveal metabolic pathway alterations in Gymnocypris przewalskii due to cold exposure
Source: BMC Genomics. 2023 Sep 14;24:545. doi: 10.1186/s12864-023-09587-9 (PMC10500822; doi:10.1186/s12864-023-09587-9)
Supplement: Supplementary file 2 — Additional file 2. [file 12864_2023_9587_MOESM2_ESM.docx]

Materials and methods of LC-MS/MS metabolite analysis

1 Sample preparation and extraction

The hepatopancreas, intestine and muscles of 12 fish (6 for 4°C and 6 for 17°C) were thawed on ice. Take 50 mg of one sample and homogenize it with 1000 ul of ice-cold methanol/water (70%, v/v). Add cold steel balls to the mixture and homogenate for at 30 Hz for 3 min. Whirl the mixture for 1 min, and then centrifuge it with 12,000 rpm at 4°C for 10 min. The collected supernatant will be used for LC-MS/MS analysis.

2 HPLC Conditions

The sample extracts were analyzed using an LC-ESI-MS/MS system (UPLC, Shim-pack UFLC SHIMADZU CBM A system, https://www.shimadzu.com/; MS, QTRAP® System, https://sciex.com/). The analytical conditions were as follows, UPLC: column, Waters ACQUITY UPLC HSS T3 C18 (1.8 µm, 2.1 mm*100 mm); column temperature, 40°C; flow rate, 0.4 ml/min; injection volume, 2μl; solvent system, water (0.04% acetic acid): acetonitrile (0.04% acetic acid); gradient program, 95:5 V/V at 0 min, 5:95 V/V at 11.0 min, 5:95 V/V at 12.0 min, 95:5 V/V at 12.1 min, 95:5 V/V at 14.0 min.

3 ESI-QTRAP-MS/MS

LIT and triple quadrupole (QQQ) scans were acquired on a triple quadrupole-linear ion trap mass spectrometer (QTRAP), QTRAP® LC-MS/MS System, equipped with an ESI Turbo Ion-Spray interface, operating in positive and negative ion mode and controlled by Analyst 1.6.3 software (Sciex). The ESI source operation parameters were as follows: source temperature 500 ∘C; ion spray voltage (IS) 5500 V (positive), -4500 V (negative); ion source gas I (GSI), gas II (GSII), and curtain gas (CUR) were set at 55, 60, and 25.0 psi, respectively; the collision gas (CAD) was high. Instrument tuning and mass calibration were performed with 10 and 100 μmol/L polypropylene glycol solutions in QQQ and LIT modes, respectively. A specific set of MRM transitions were monitored for each period according to the metabolites eluted within this period.

4 Metabolite profiling.

Metabolite profiling was carried out using a widely targeted metabolome method by Wuhan Metware Biotechnology Co., Ltd. (Wuhan, China) (http://www.metware.cn/). The sample were extracted as previously described before analysis using an LC–electrospray ionization (ESI)-MS/MS system. The extracts were absorbed (CNWBOND Carbon-GCB SPE Cartridge, 250 mg, 3 ml; Shanghai ANPEL Scientific Instrument Co., Ltd.). Quantification of metabolites was carried out using a multiple reaction monitoring (MRM) method with 6 biological repetitions each temperature treatment.

5 Bioinformatic analysis

The principal component analysis (PCA) was performed by statistics function prcomp within R (www.r-project.org). The data was unit variance scaled before unsupervised PCA. The standardized scores of the first three components which explained the highest variation were used to make biplots. The hierarchical cluster analysis (HCA) results of samples and metabolites were presented as heatmaps with dendrograms, while pearson correlation coefficients (PCC) between samples were caculated by the cor function in R and presented by heatmaps. Both HCA and PCC were carried out by R package pheatmap. For HCA, normalized signal intensities of metabolites (unit variance scaling) are visualized as a color spectrum. Significantly regulated metabolites between groups were determined by VIP >= 1 and absolute Log2FC (fold change) >= 1. VIP values were extracted from OPLS-DA result, which also contain score plots and permutation plots, was generated using R package MetaboAnalystR. The data was log transform (log2) and mean centering before OPLS-DA. In order to avoid overfitting, a permutation test (200 permutations) was performed. Identified metabolites were annotated using KEGG Compound database (http://www.kegg.jp/kegg/compound/), annotated metabolites were then mapped to KEGG Pathway database (http://www.kegg.jp/kegg/pathway.html). Pathways with significantly regulated metabolites mapped to were then fed into MSEA (metabolite sets enrichment analysis), their significance was determined by hypergeometric test’s p-values.
