# Supplementary figures and images for: Physiological, metabolomic, and transcriptomic reveal metabolic pathway alterations in Gymnocypris przewalskii due to cold exposure
Source: BMC Genomics. 2023 Sep 14;24:545. doi: 10.1186/s12864-023-09587-9 (PMC10500822; doi:10.1186/s12864-023-09587-9)

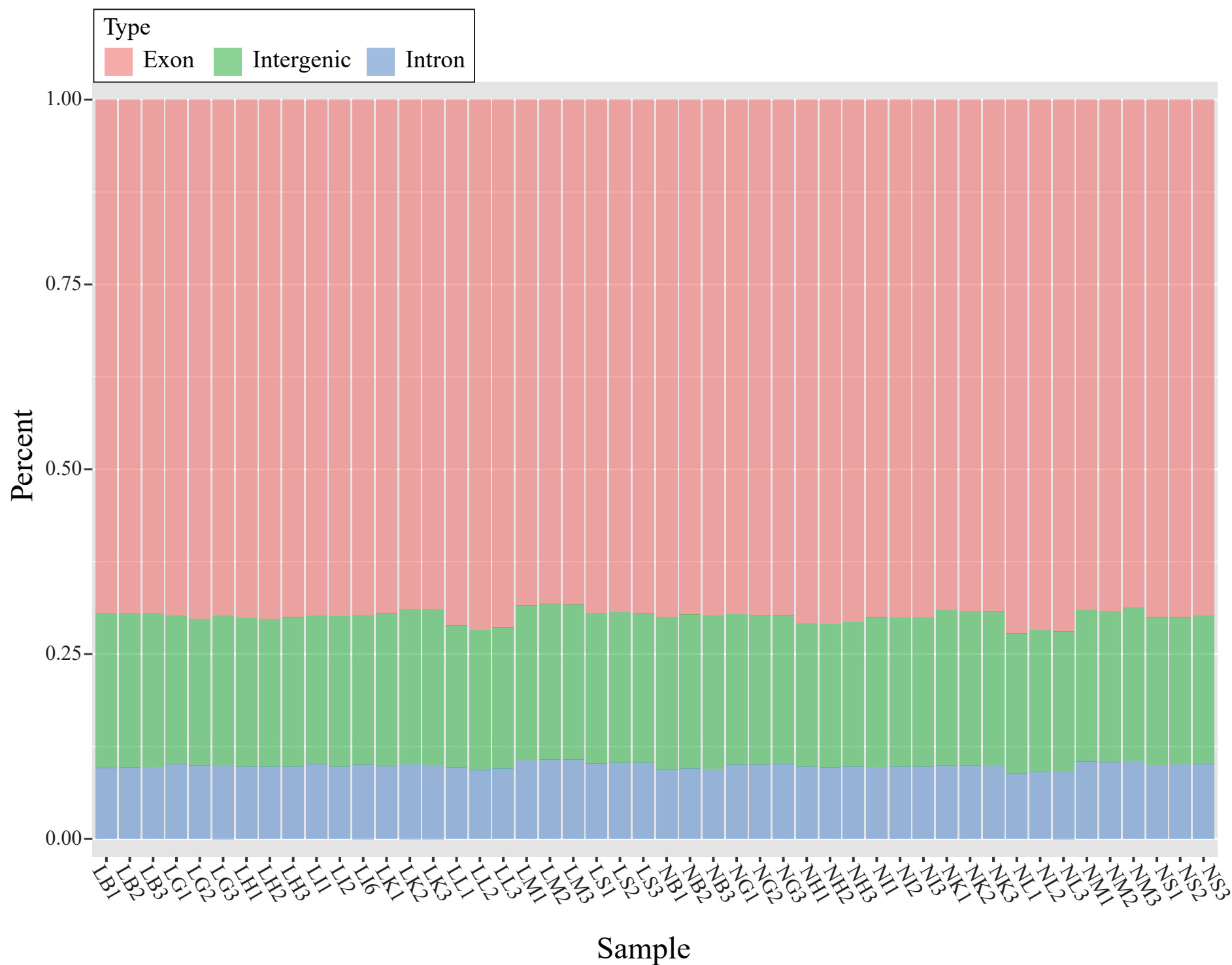

Supplement: Supplementary file 5 — Additional file 5. [file 12864_2023_9587_MOESM5_ESM.pdf]

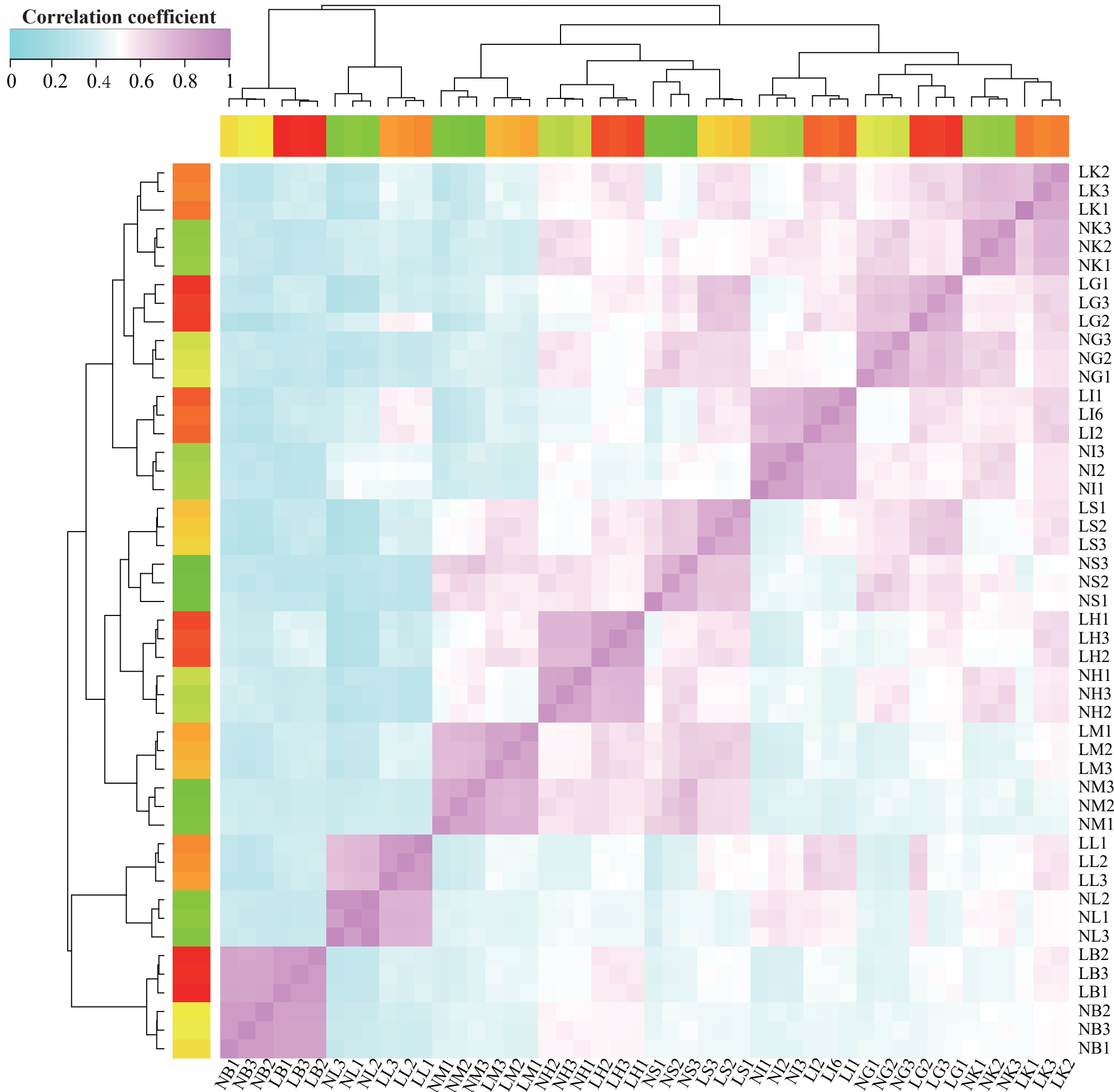

Supplement: Supplementary file 6 — Additional file 6. [file 12864_2023_9587_MOESM6_ESM.pdf]

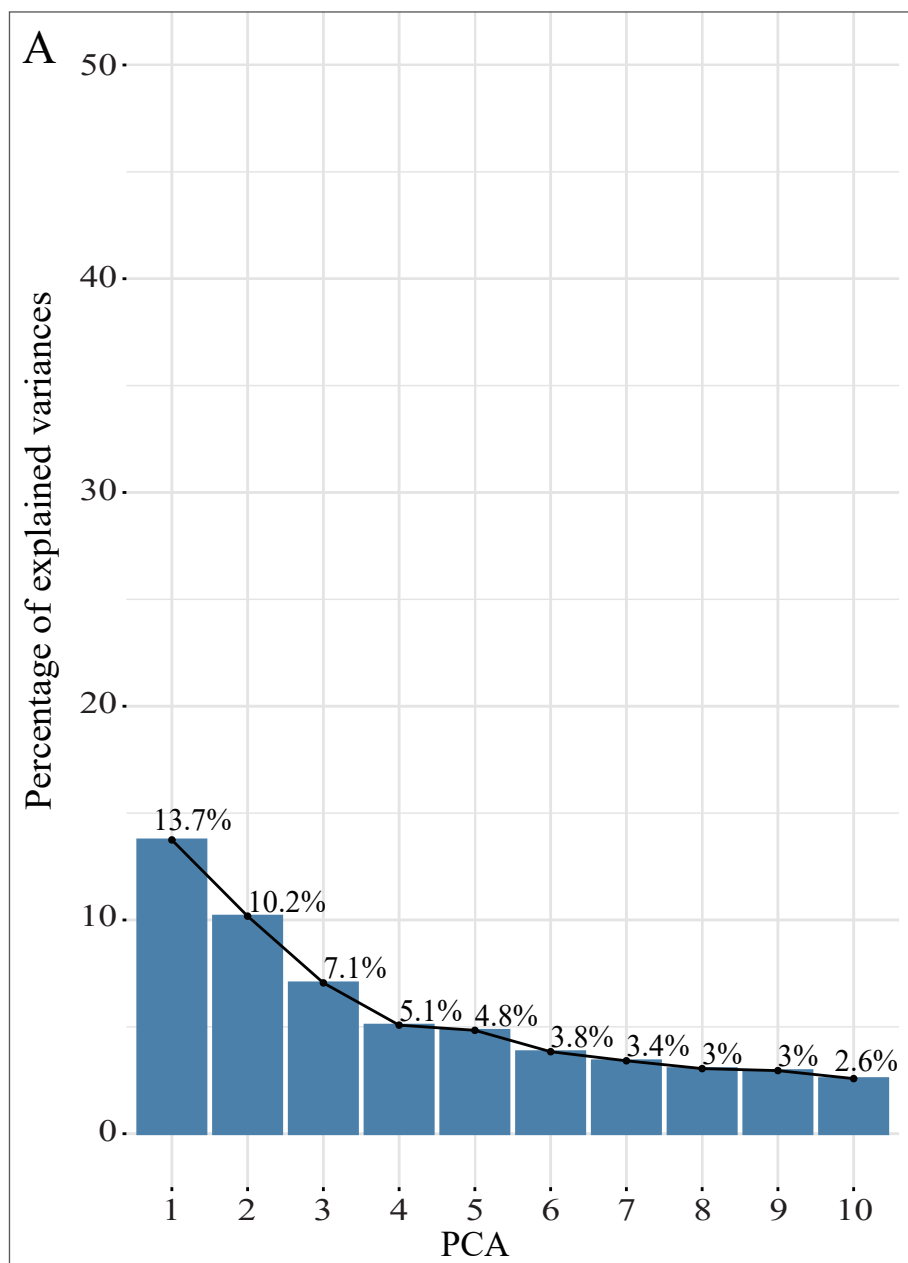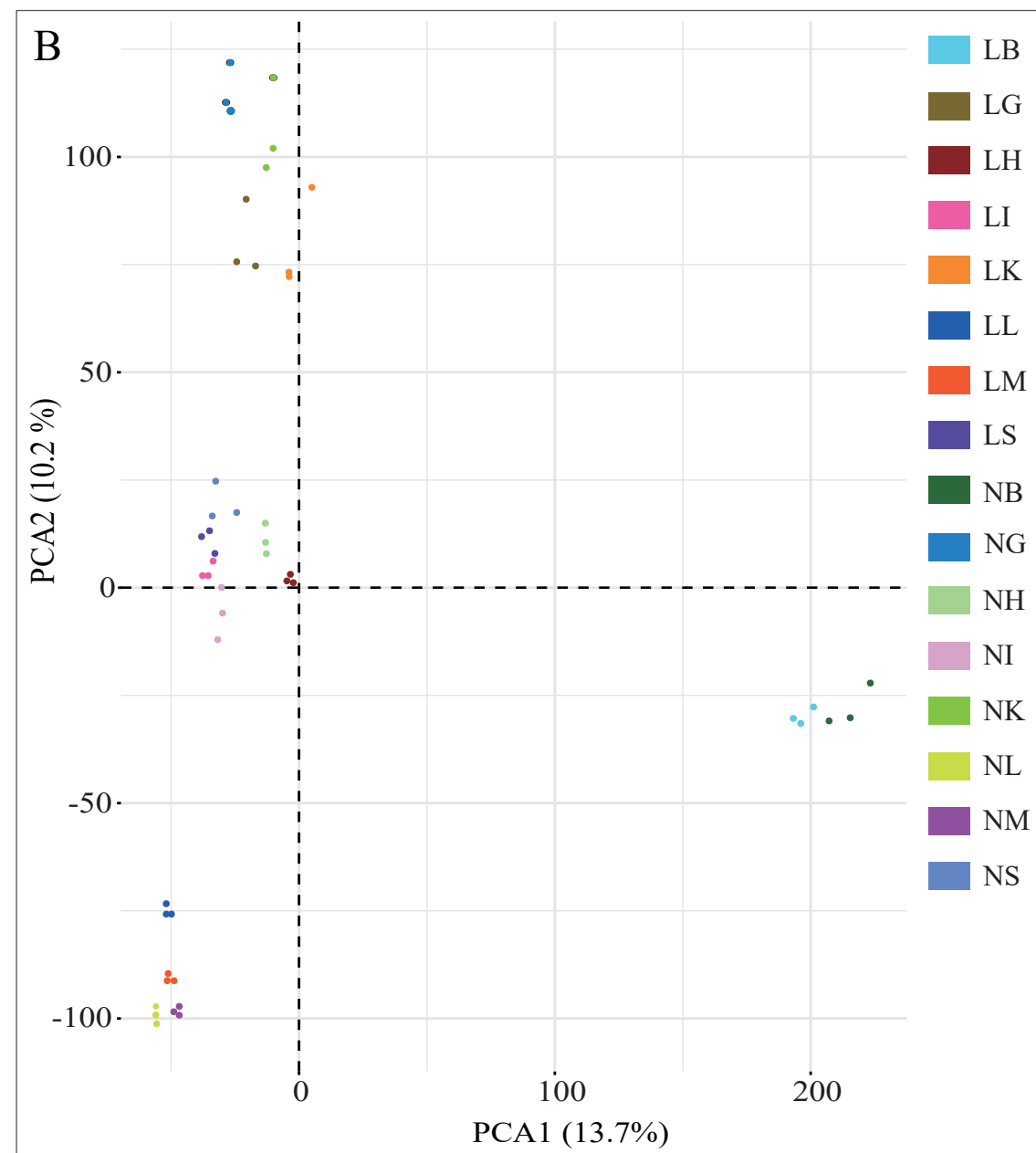

Supplement: Supplementary file 7 — Additional file 7. [file 12864_2023_9587_MOESM7_ESM.pdf]

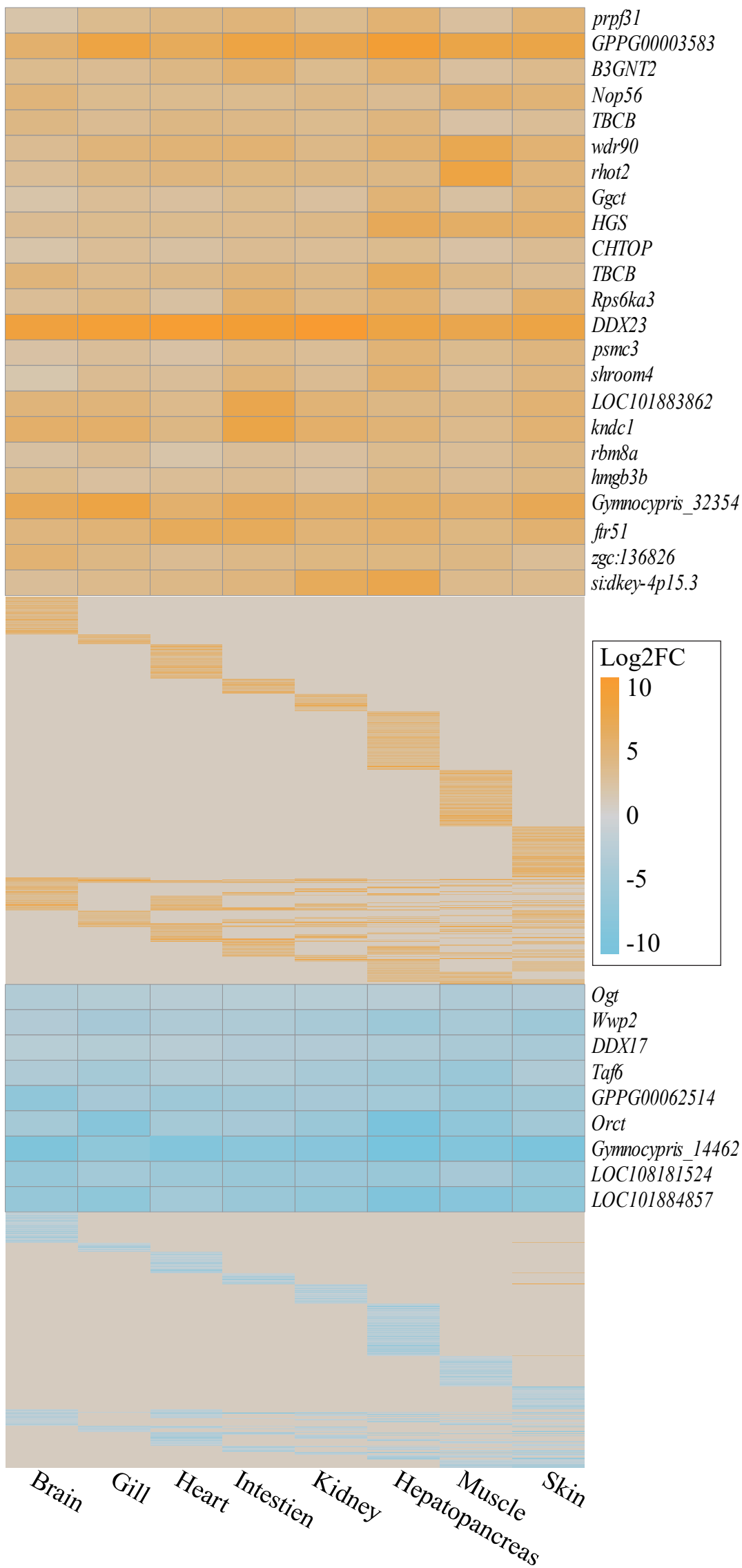

Supplement: Supplementary file 8 — Additional file 8. [file 12864_2023_9587_MOESM8_ESM.pdf]

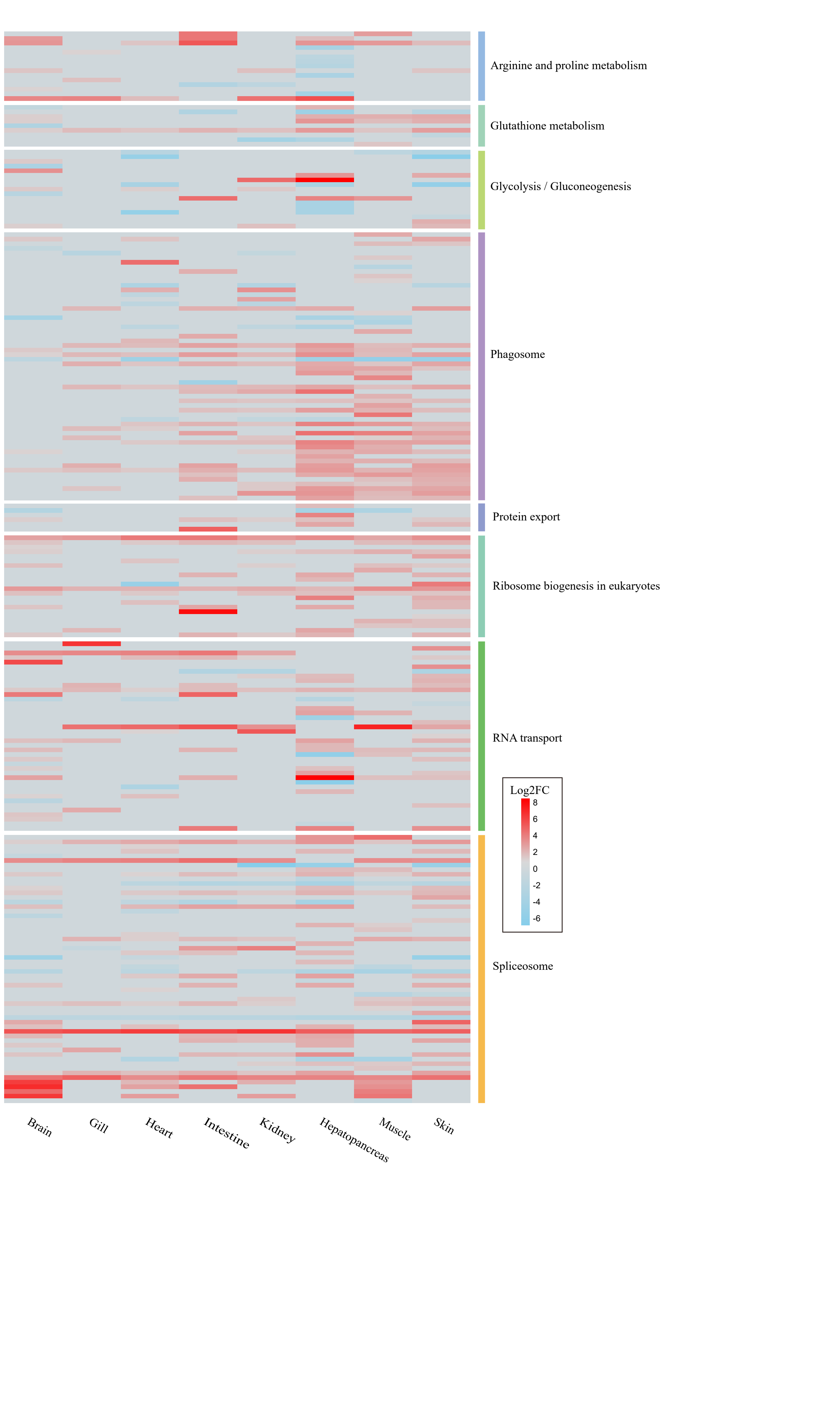

Supplement: Supplementary file 9 — Additional file 9. [file 12864_2023_9587_MOESM9_ESM.pdf]

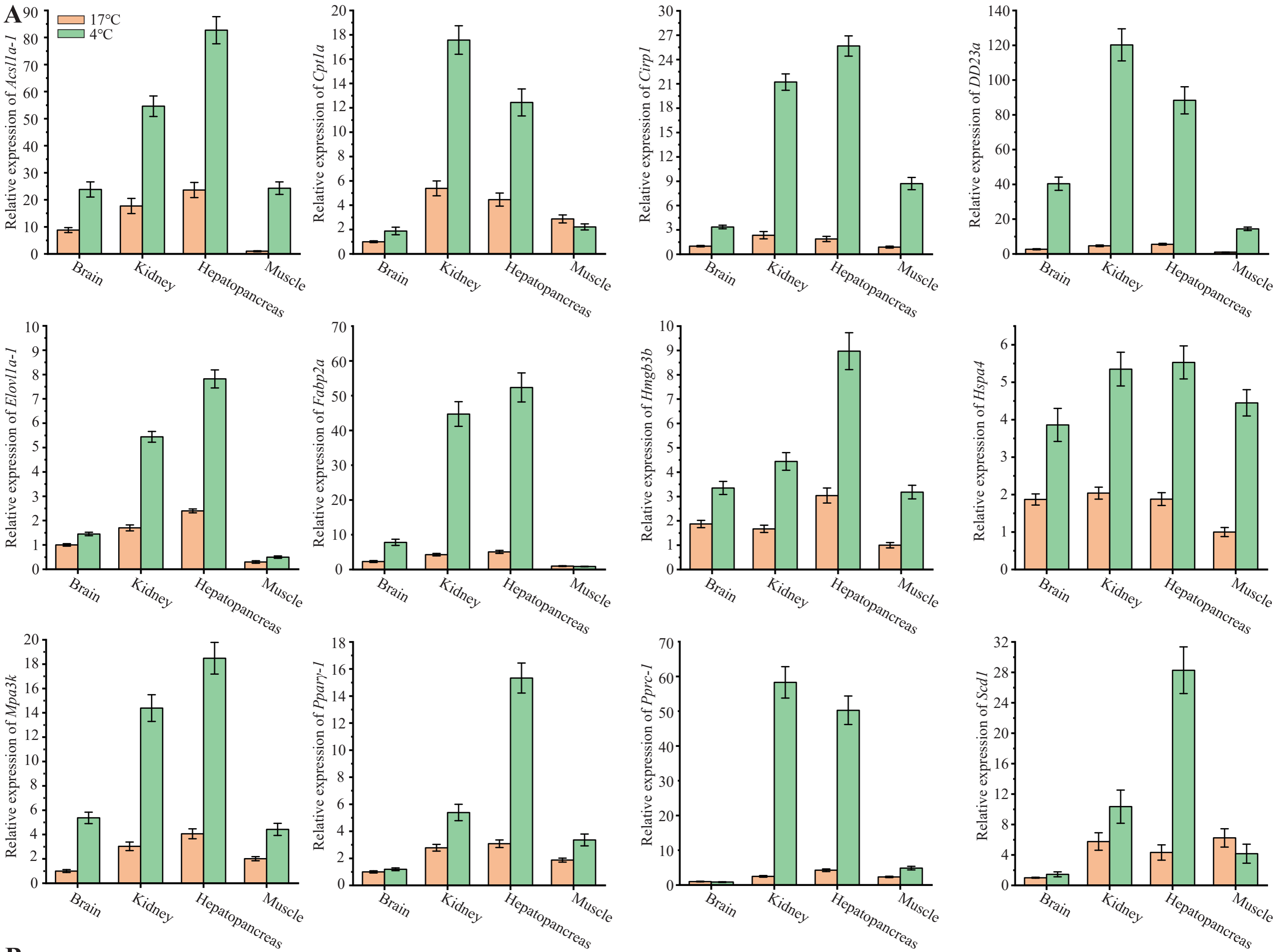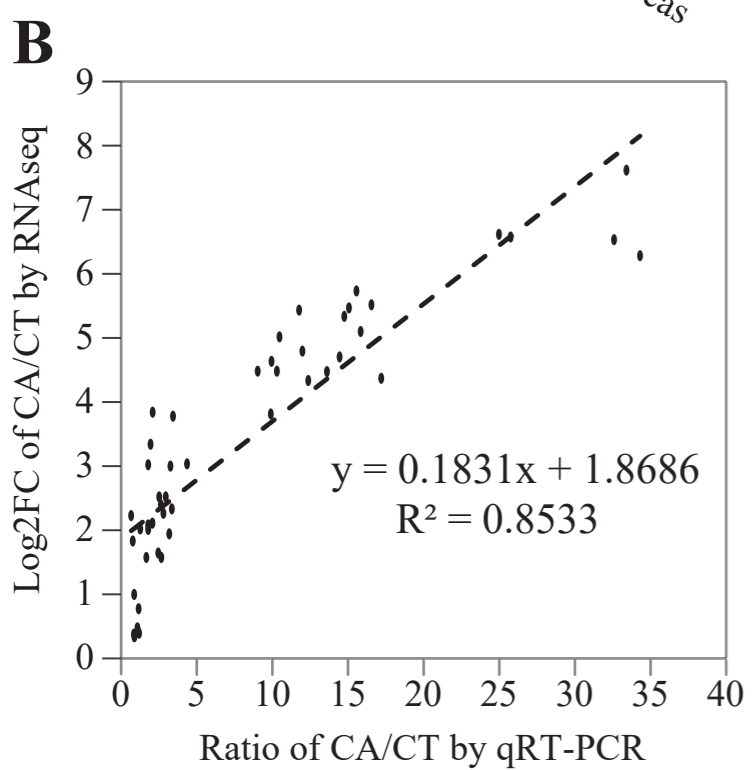

Supplement: Supplementary file 10 — Additional file 10. [file 12864_2023_9587_MOESM10_ESM.pdf]

17 °C 4 °C

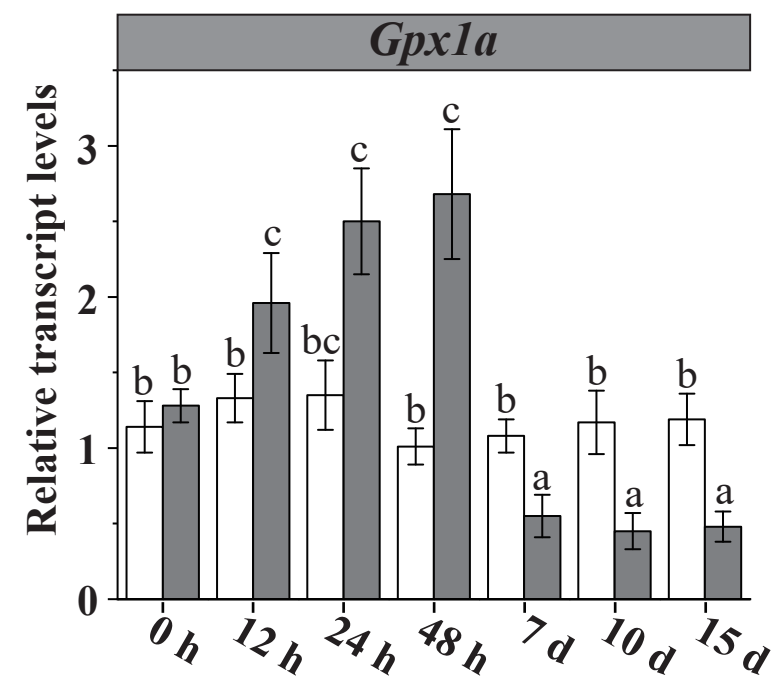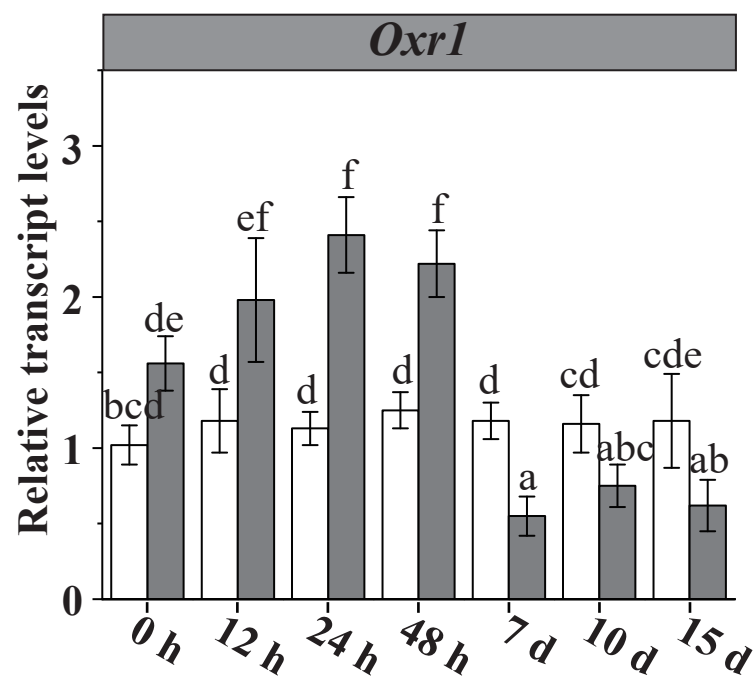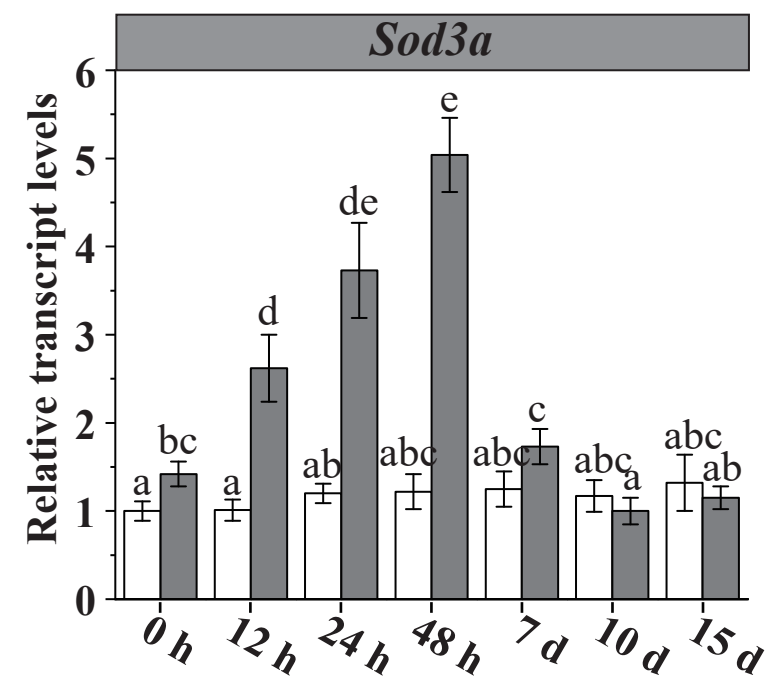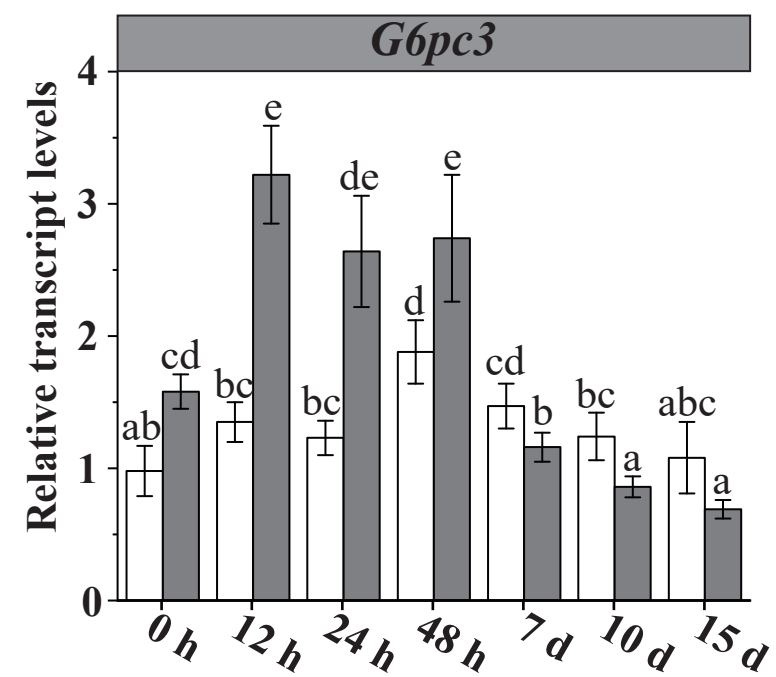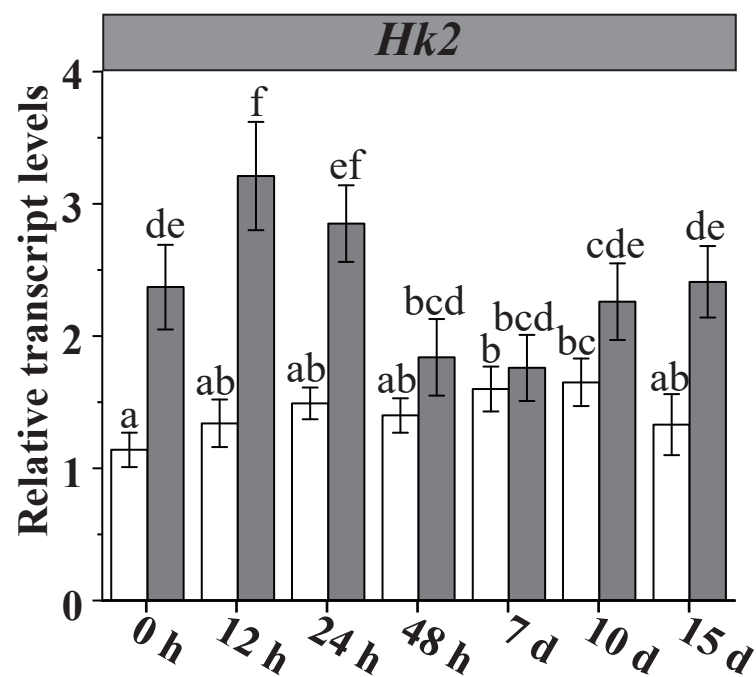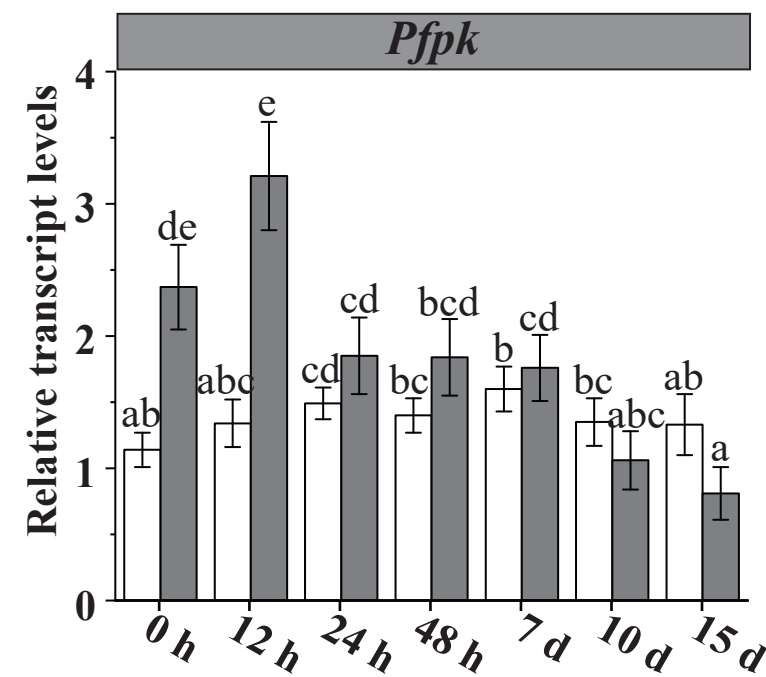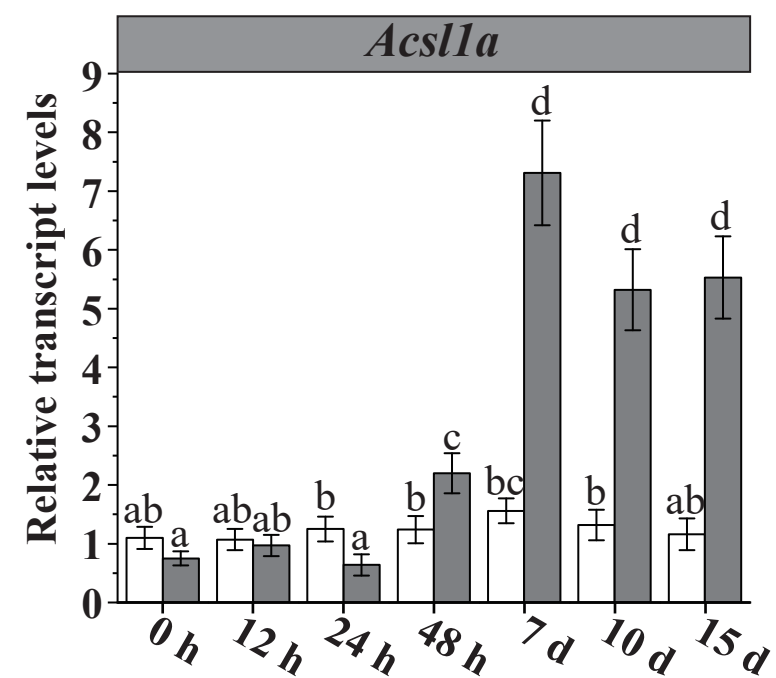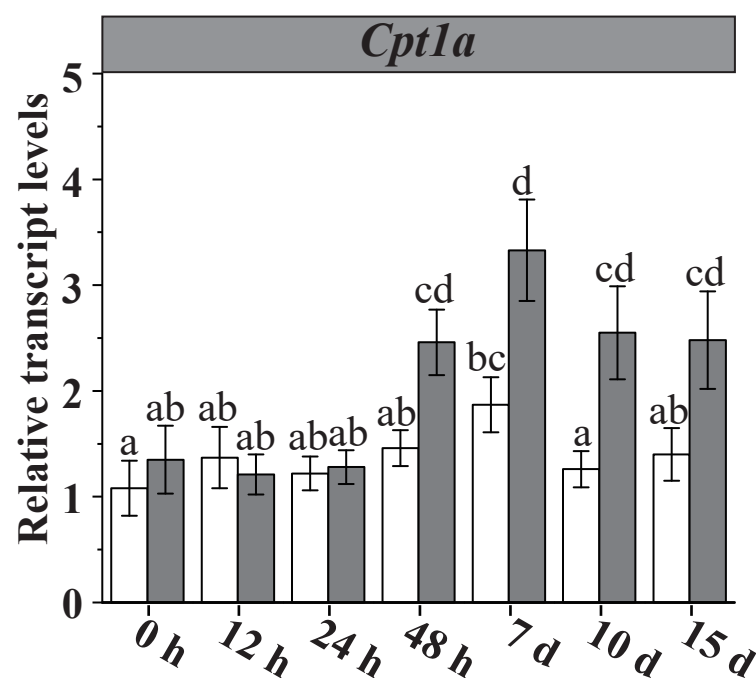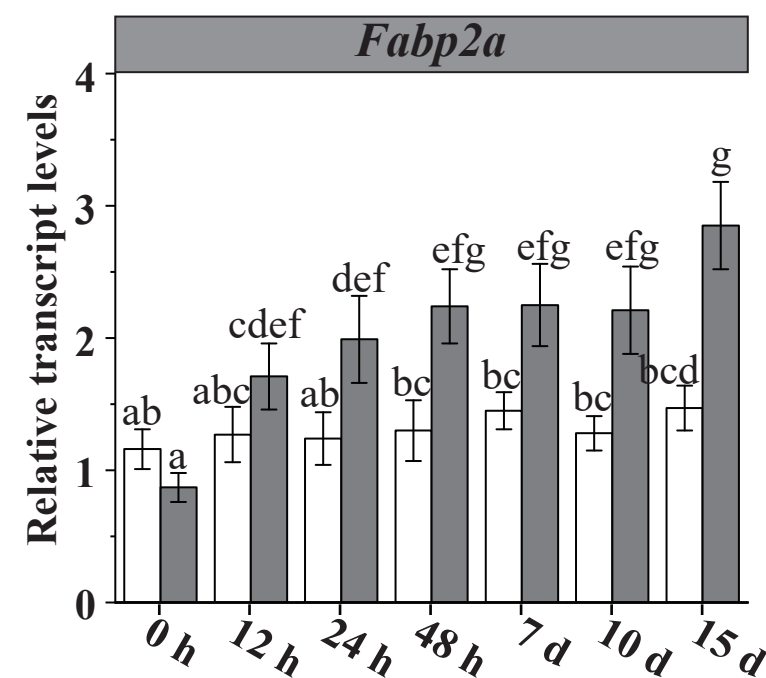

Supplement: Supplementary file 11 — Additional file 11. [file 12864_2023_9587_MOESM11_ESM.pdf]

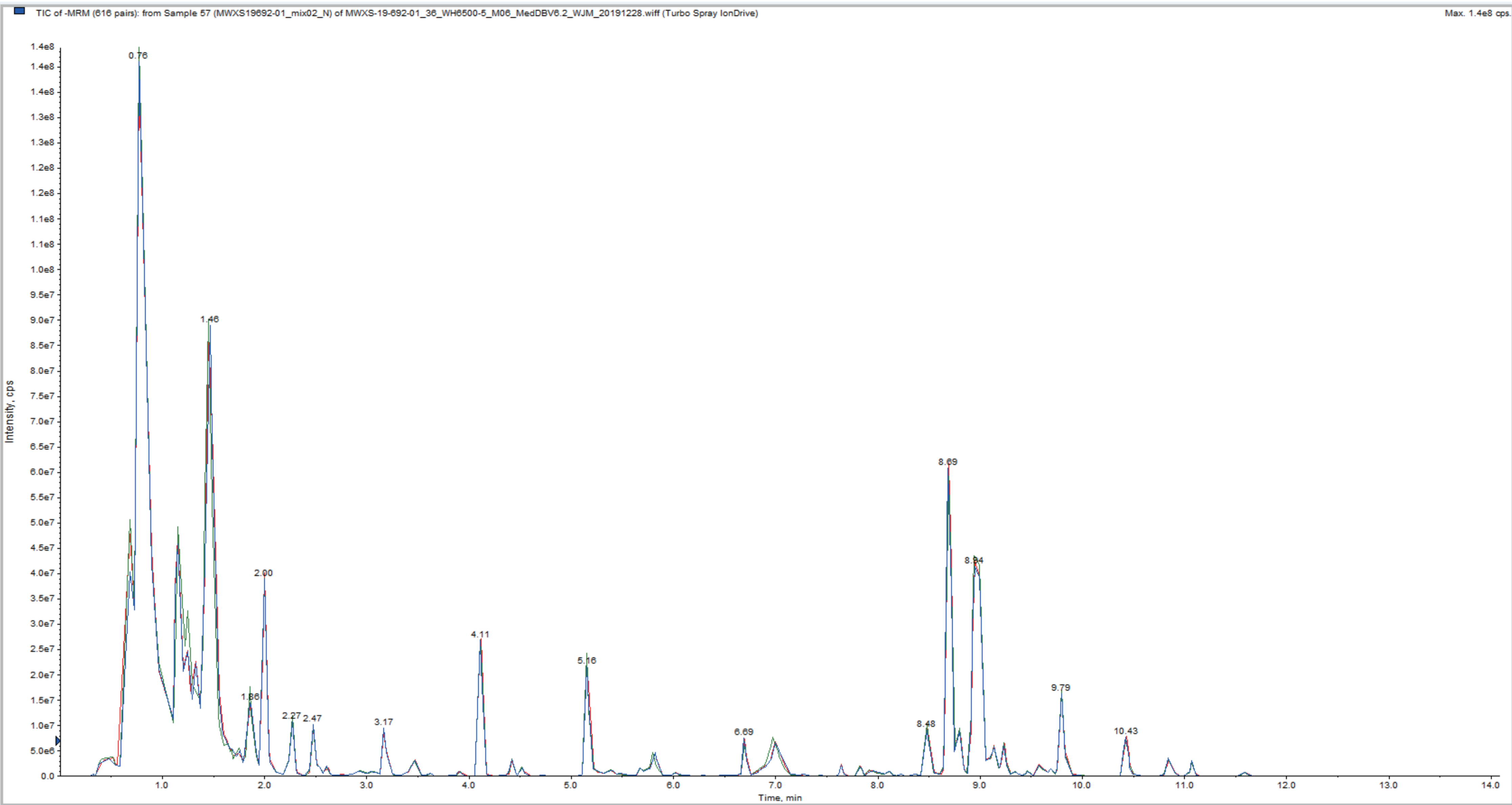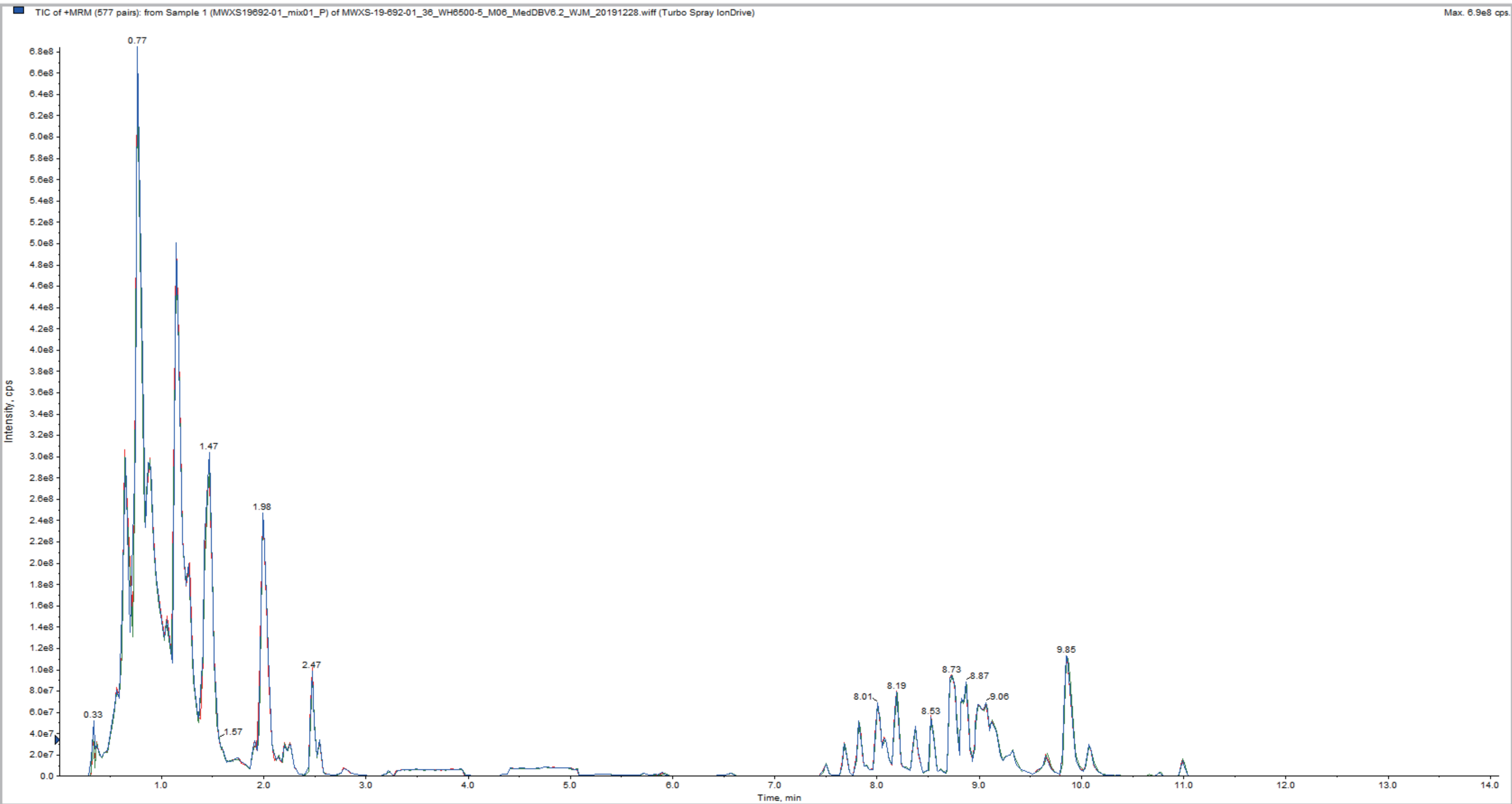

Supplement: Supplementary file 12 — Additional file 12. [file 12864_2023_9587_MOESM12_ESM.pdf]

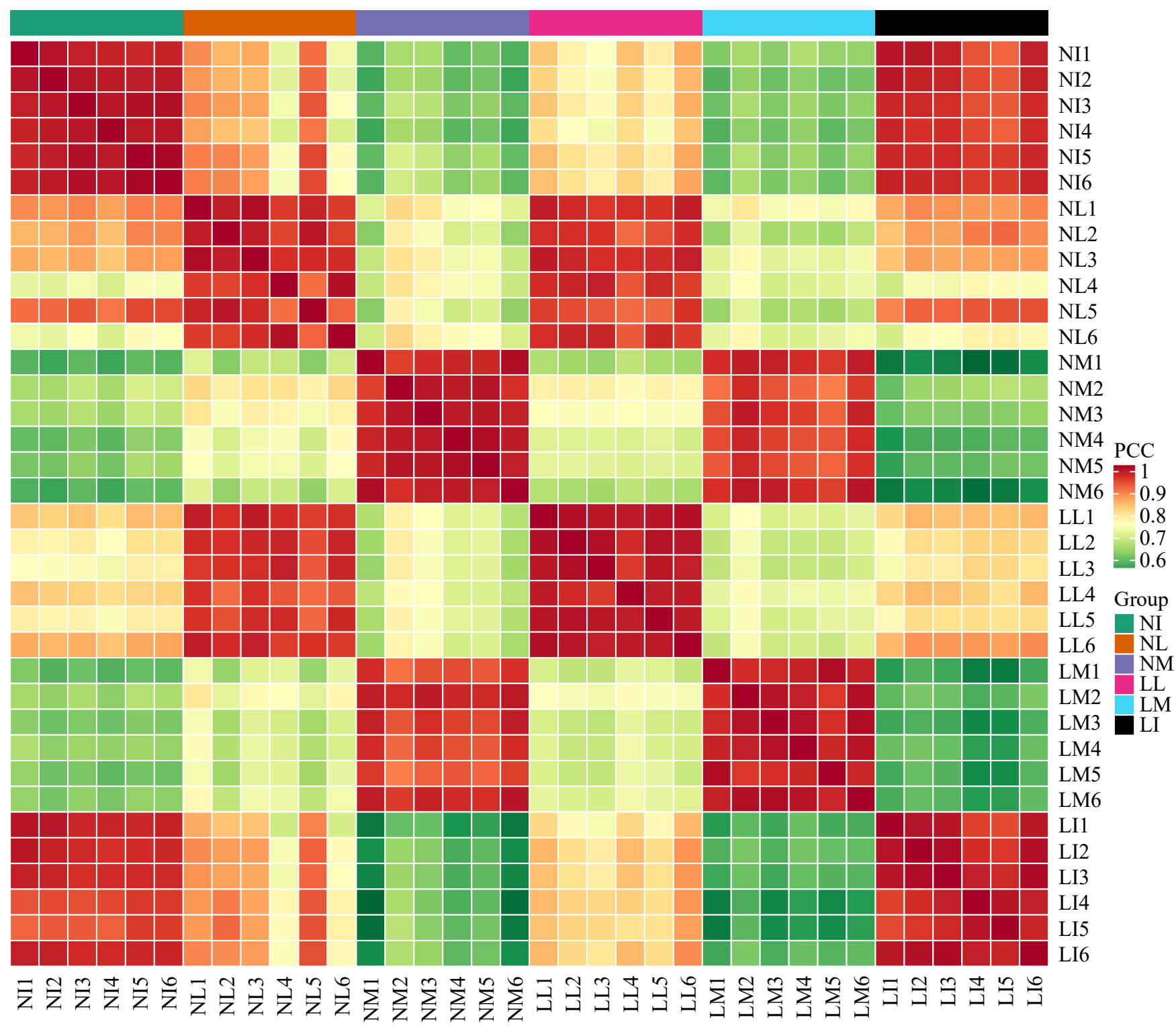

Supplement: Supplementary file 13 — Additional file 13. [file 12864_2023_9587_MOESM13_ESM.pdf]

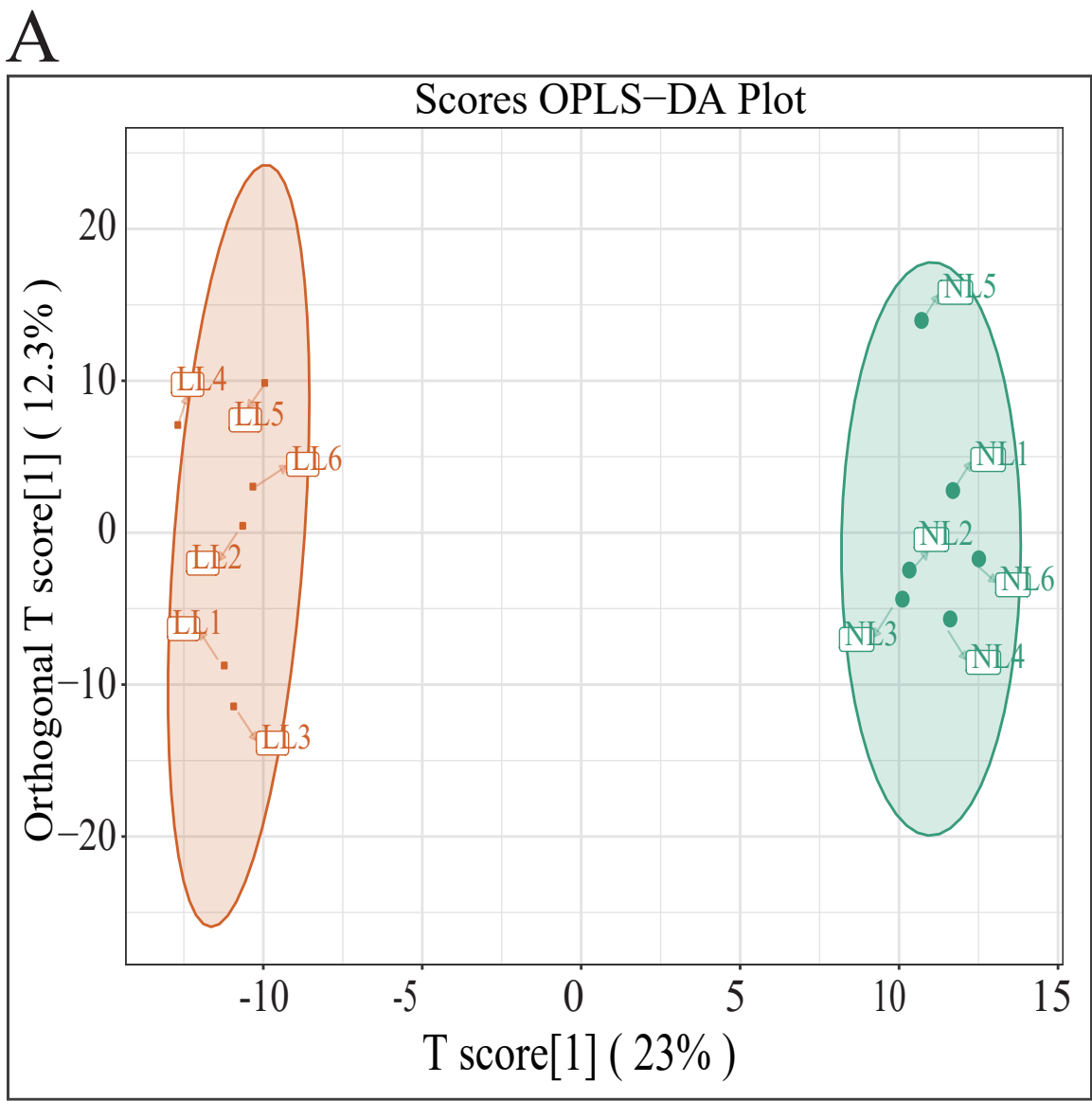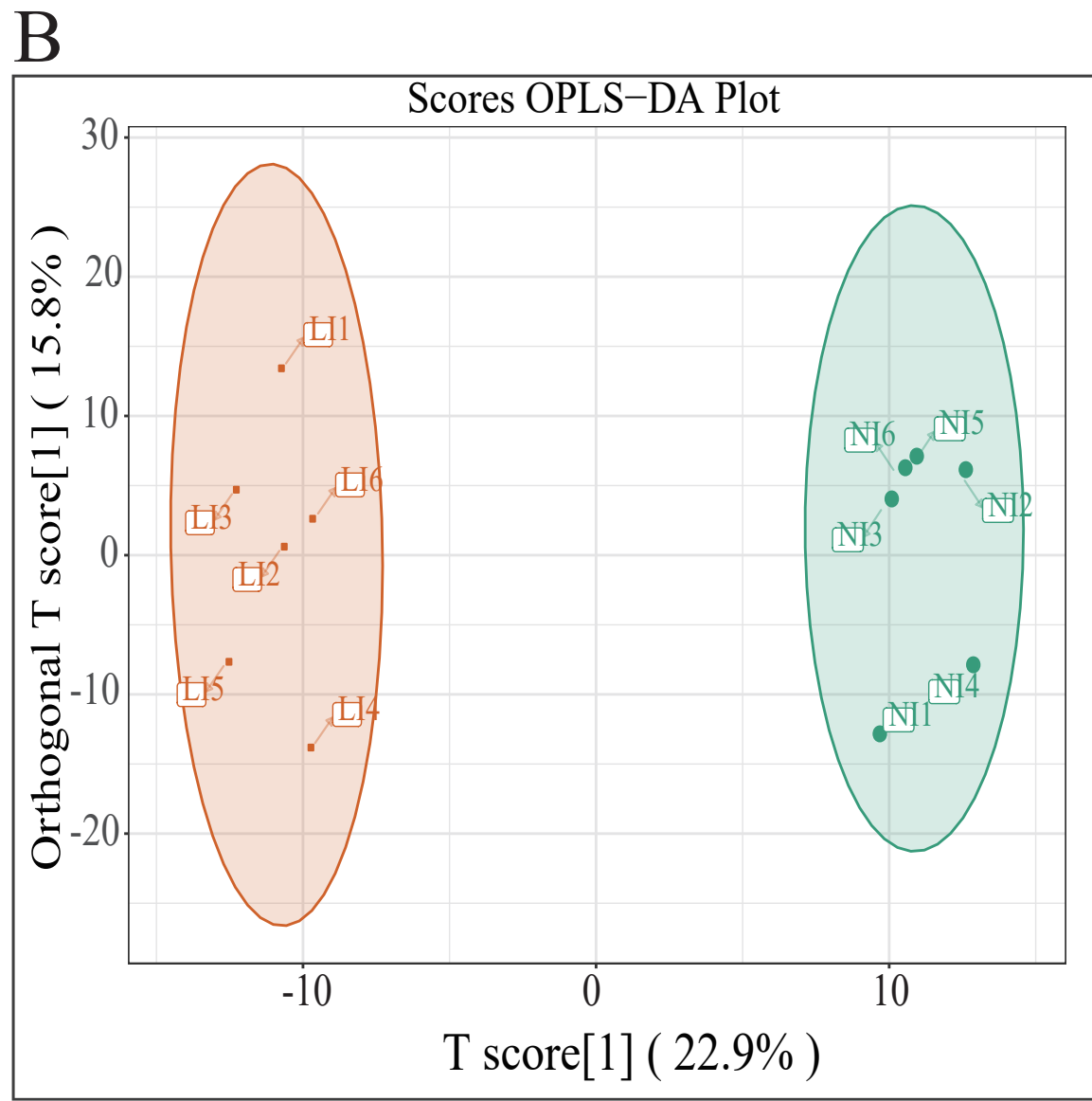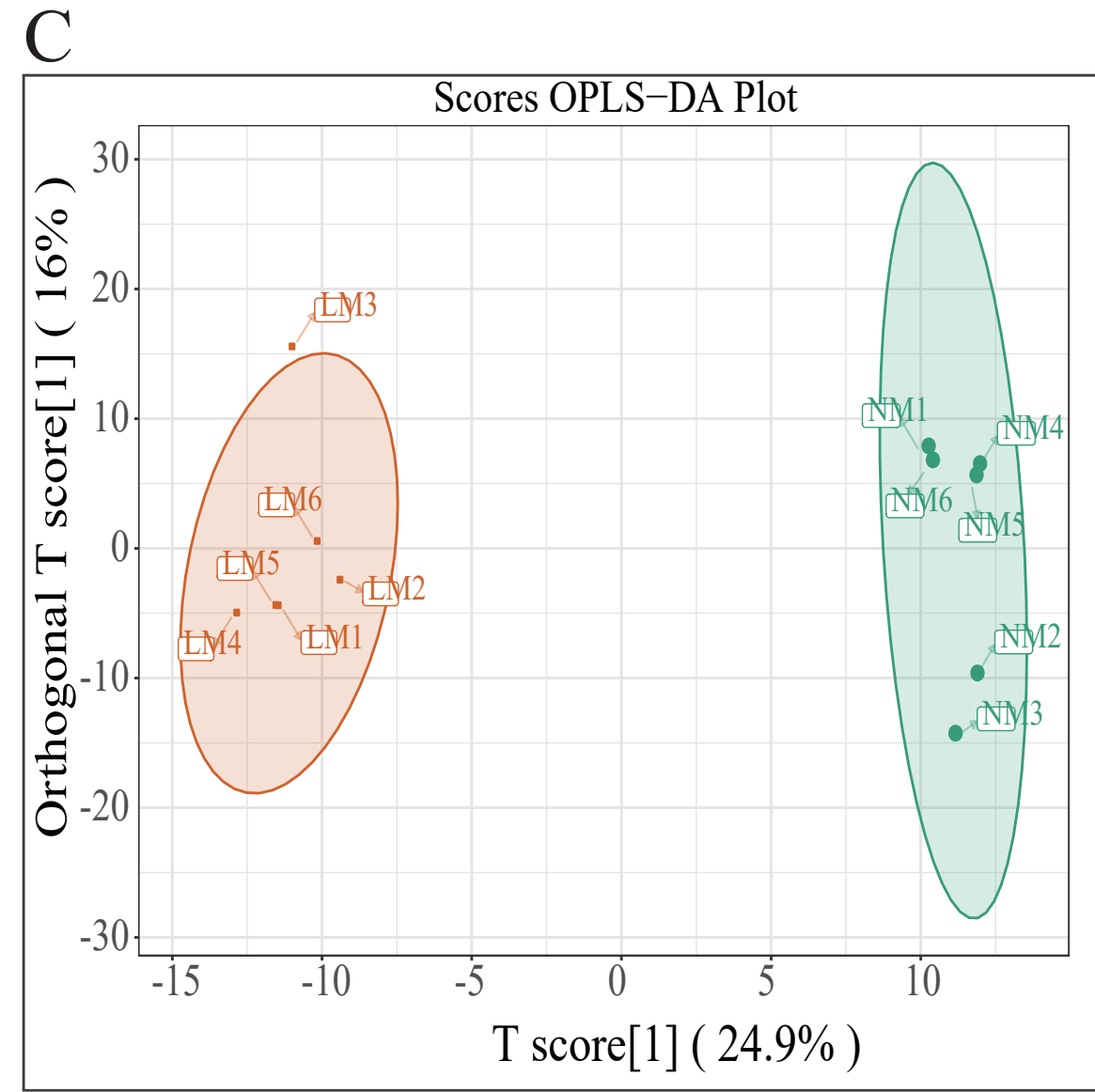

Supplement: Supplementary file 15 — Additional file 15. [file 12864_2023_9587_MOESM15_ESM.pdf]

# DEMs in the hepatopancreas

DEGs in the hepatopancreas

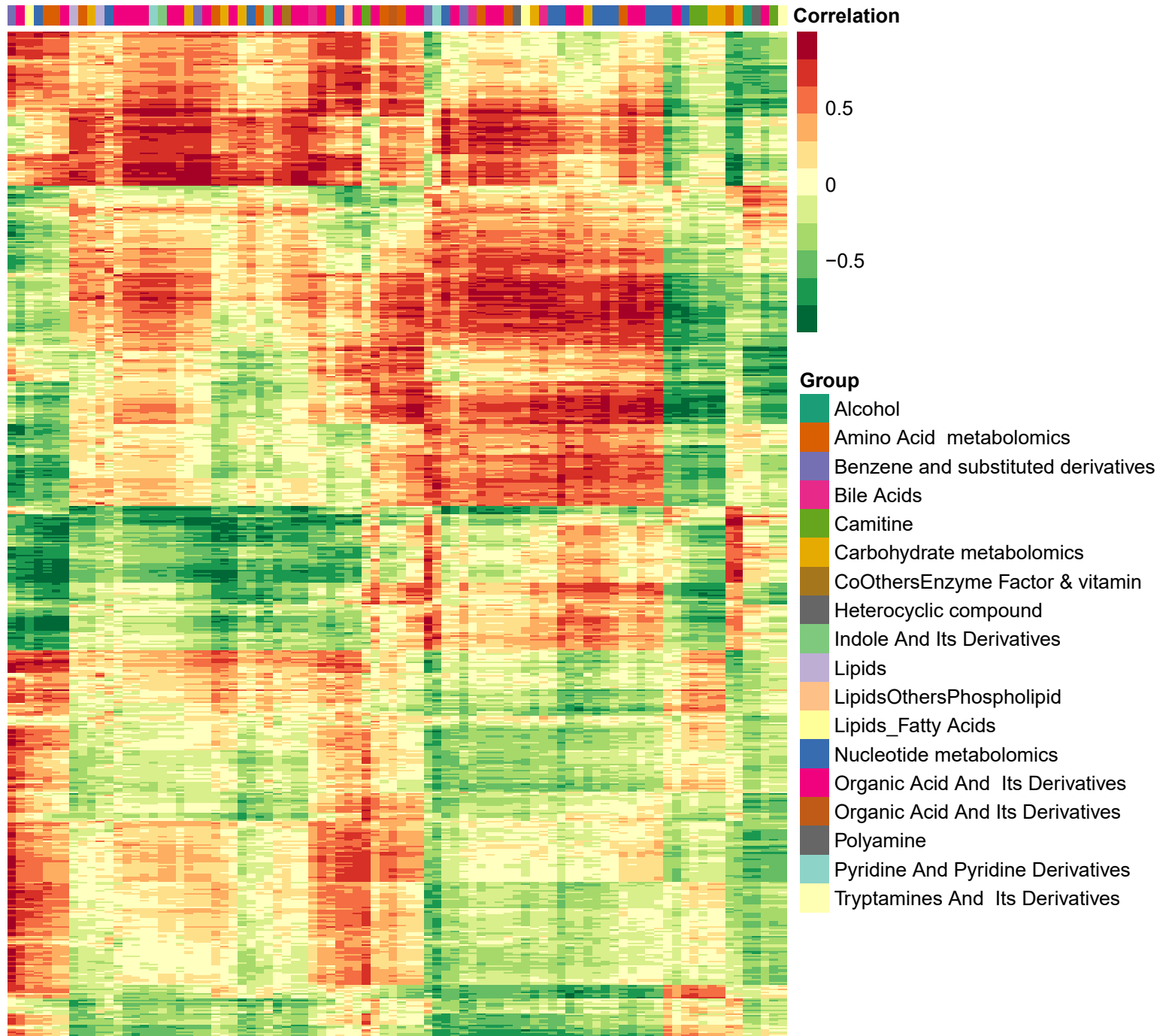

Supplement: Supplementary file 16 — Additional file 16. [file 12864_2023_9587_MOESM16_ESM.pdf]

# DEMs in the intestine

DEGs in the intestine

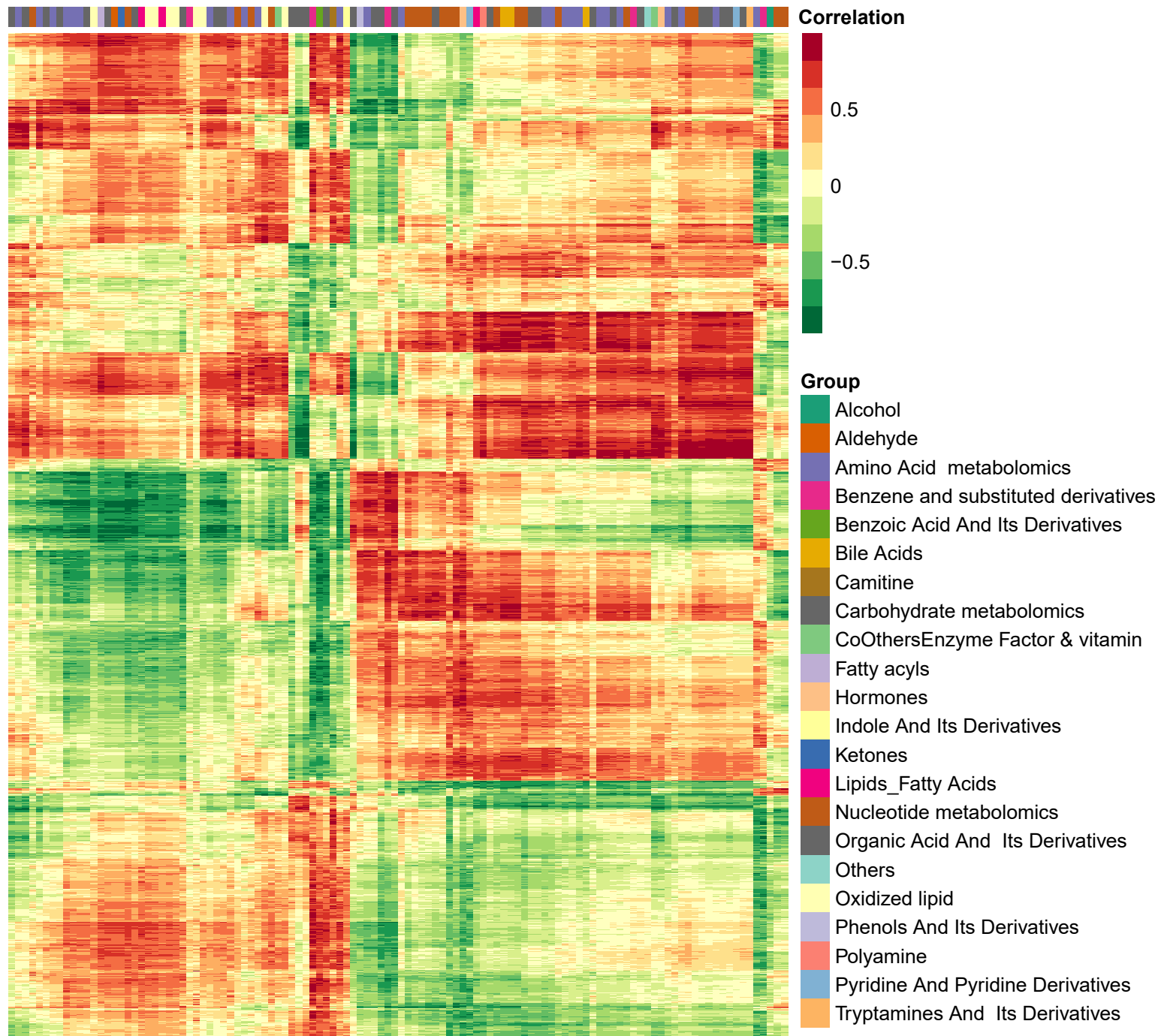

Supplement: Supplementary file 17 — Additional file 17. [file 12864_2023_9587_MOESM17_ESM.pdf]

## DEMs in the muscle

DEGs in the muscle

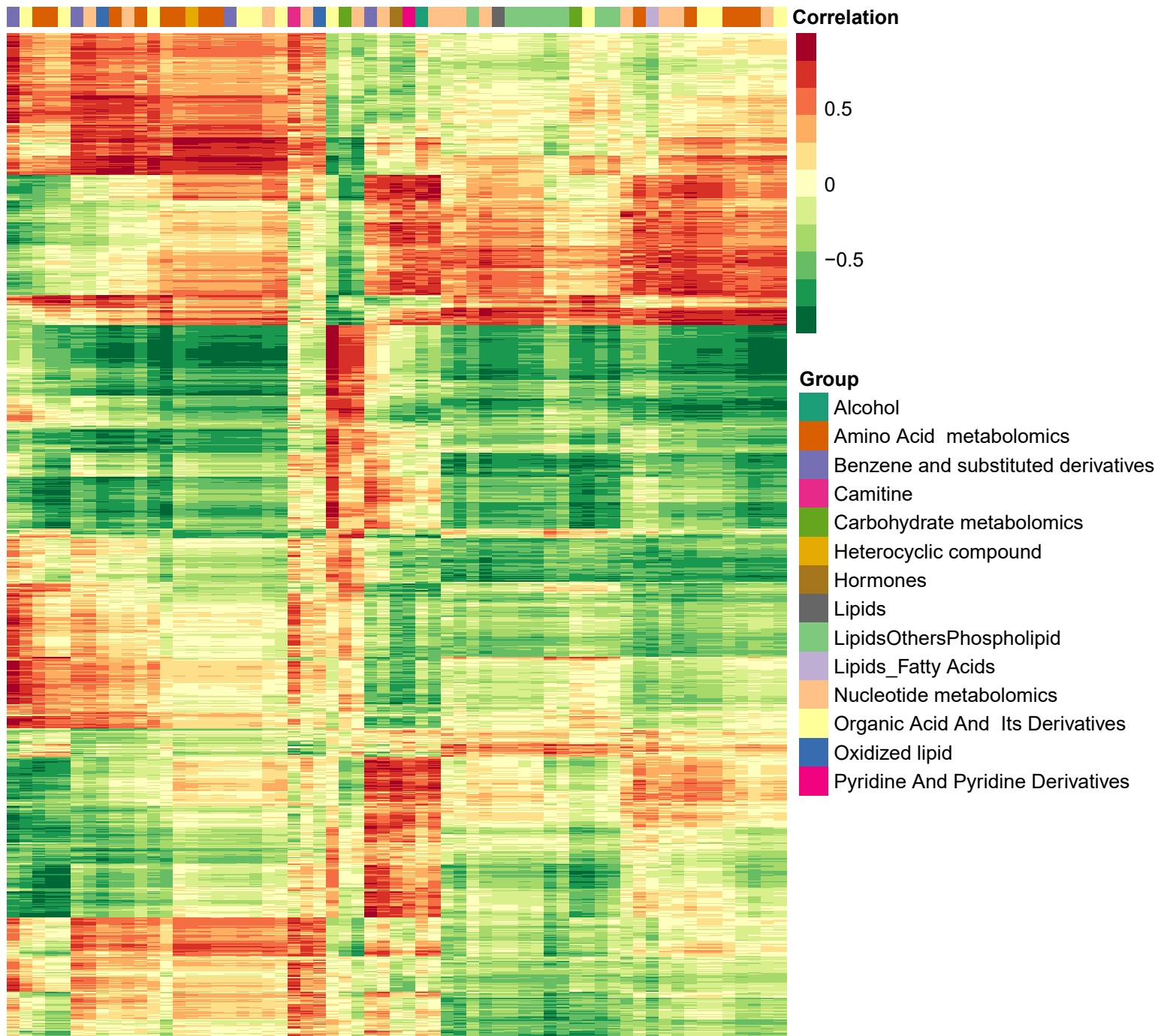

Supplement: Supplementary file 18 — Additional file 18. [file 12864_2023_9587_MOESM18_ESM.pdf]
