# Supplementary material for: Physiological, metabolomic, and transcriptomic reveal metabolic pathway alterations in Gymnocypris przewalskii due to cold exposure
Source: BMC Genomics. 2023 Sep 14;24:545. doi: 10.1186/s12864-023-09587-9 (PMC10500822; doi:10.1186/s12864-023-09587-9)
Supplement: Supplementary file 14 — Additional file 14. [file 12864_2023_9587_MOESM14_ESM.pdf]

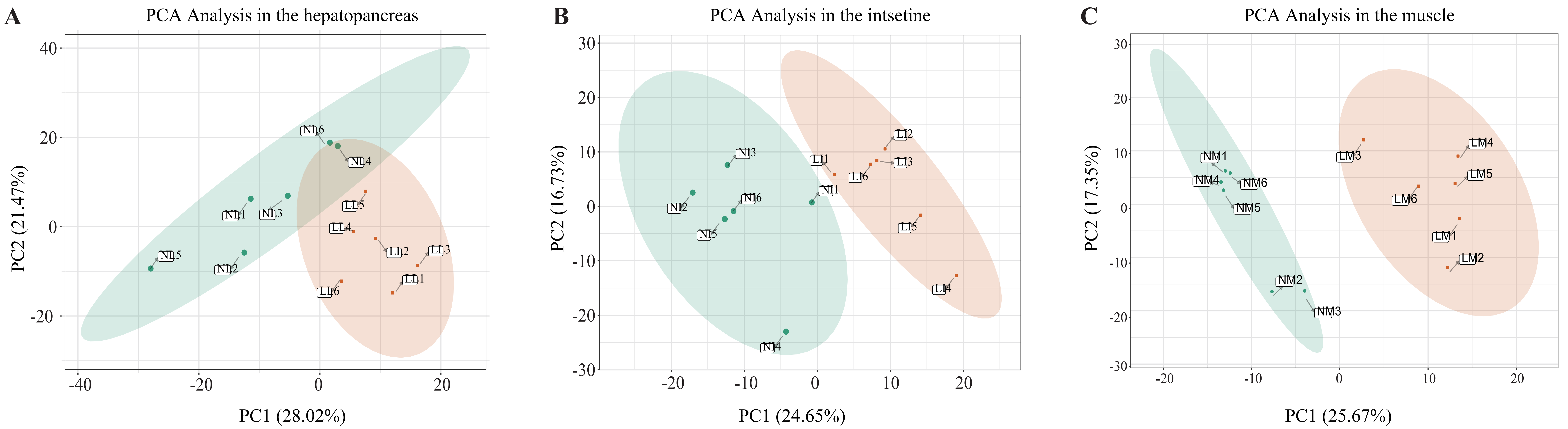

A
PCA Analysis in the hepatopancreas
PC2 (21.47%)
PC1 (28.02%)
40
20
0
-20
-40
-20
0
20
B
PCA Analysis in the intsetine
PC2 (16.73%)
PC1 (24.65%)
30
20
10
0
-10
-20
-30
-20
-10
0
10
20
C
PCA Analysis in the muscle
PC2 (17.35%)
PC1 (25.67%)
30
20
10
0
-10
-20
-30
-20
-10
0
10
20
NM1
NM4
NM6
NM5
NM2
NM3
LM3
LM4
LM5
LM6
LM1
LM2
