# Supplementary material for: Physiological, metabolomic, and transcriptomic reveal metabolic pathway alterations in Gymnocypris przewalskii due to cold exposure
Source: BMC Genomics. 2023 Sep 14;24:545. doi: 10.1186/s12864-023-09587-9 (PMC10500822; doi:10.1186/s12864-023-09587-9)
Supplement: Supplementary file 19 — Additional file 19. [file 12864_2023_9587_MOESM19_ESM.pdf]

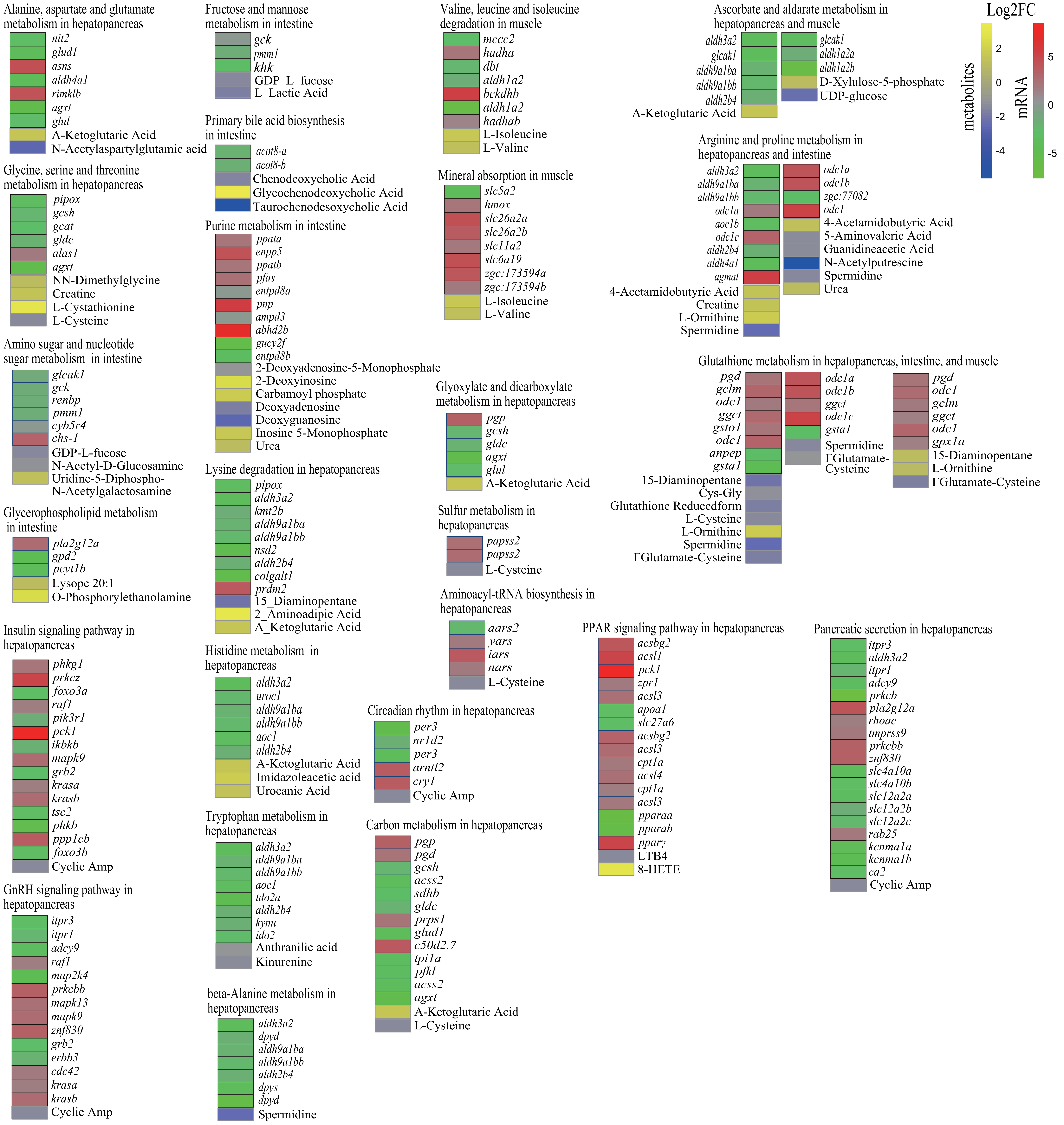
Log2FC
metabolites
2
0
-2
-4
mRNA
5
0
-5
Alanine, aspartate and glutamate metabolism in hepatopancreas
nit2
glud1
asns
aldh4a1
rimklb
agxt
glul
A-Ketoglutaric Acid
N-Acetylaspartylglutamic acid
Glycine, serine and threonine metabolism in hepatopancreas
pipox
gcsh
gcat
gldc
alas1
agxt
NN-Dimethylglycine
Creatine
L-Cystathionine
L-Cysteine
Amino sugar and nucleotide sugar metabolism in intestine
glcak1
gck
renbp
pmm1
cyb5r4
chs-1
GDP-L-fucose
N-Acetyl-D-Glucosamine
Uridine-5-Diphospho-N-Acetylgalactosamine
Glycerophospholipid metabolism in intestine
pla2g12a
gpd2
pcyt1b
Lysopc 20:1
O-Phosphorylethanolamine
Insulin signaling pathway in hepatopancreas
phkg1
prkcz
foxo3a
raf1
pik3r1
pck1
ikbkb
mapk9
grb2
krasa
krasb
tsc2
phkb
ppp1cb
foxo3b
Cyclic Amp
GnRH signaling pathway in hepatopancreas
itpr3
itpr1
adcy9
raf1
map2k4
prkcbb
mapk13
mapk9
znf830
grb2
erbb3
cdc42
krasa
krasb
Cyclic Amp
Fructose and mannose metabolism in intestine
gck
pmm1
khk
GDP_L_fucose
L_Lactic Acid
Primary bile acid biosynthesis in intestine
acot8-a
acot8-b
Chenodeoxycholic Acid
Glycochenodeoxycholic Acid
Taurochenodesoxycholic Acid
Purine metabolism in intestine
ppata
enpp5
ppatb
pfas
entpd8a
pnp
ampd3
abhd2b
gucy2f
entpd8b
2-Deoxyadenosine-5-Monophosphate
2-Deoxyinosine
Carbamoyl phosphate
Deoxyadenosine
Deoxyguanosine
Inosine 5-Monophosphate
Urea
Lysine degradation in hepatopancreas
pipox
aldh3a2
kmt2b
aldh9a1ba
aldh9a1bb
nsd2
aldh2b4
colgalt1
prdm2
15_Diaminopentane
2_Aminoadipic Acid
A_Ketoglutaric Acid
Histidine metabolism in hepatopancreas
aldh3a2
uroc1
aldh9a1ba
aldh9a1bb
aoc1
aldh2b4
A-Ketoglutaric Acid
Imidazoleacetic acid
Urocanic Acid
Tryptophan metabolism in hepatopancreas
aldh3a2
aldh9a1ba
aldh9a1bb
aoc1
tdo2a
aldh2b4
kynu
ido2
Anthranilic acid
Kinurenine
beta-Alanine metabolism in hepatopancreas
aldh3a2
dpyd
aldh9a1ba
aldh9a1bb
aldh2b4
dpys
dpyd
Spermidine
Valine, leucine and isoleucine degradation in muscle
mccc2
hadha
dbt
aldh1a2
bckdhb
aldh1a2
hadhab
L-Isoleucine
L-Valine
Mineral absorption in muscle
slc5a2
hmox
slc26a2a
slc26a2b
slc11a2
slc6a19
zgc:173594a
zgc:173594b
L-Isoleucine
L-Valine
Glyoxylate and dicarboxylate metabolism in hepatopancreas
pgp
gcsh
gldc
agxt
glul
A-Ketoglutaric Acid
Sulfur metabolism in hepatopancreas
papss2
papss2
L-Cysteine
Aminoacyl-tRNA biosynthesis in hepatopancreas
aars2
yars
iars
nars
L-Cysteine
Circadian rhythm in hepatopancreas
per3
nr1d2
per3
arntl2
cry1
Cyclic Amp
Carbon metabolism in hepatopancreas
pgp
pgd
gcsh
acss2
sdhb
gldc
prps1
glud1
c50d2.7
tpi1a
pfkl
acss2
agxt
A-Ketoglutaric Acid
L-Cysteine
Ascorbate and aldarate metabolism in hepatopancreas and muscle
aldh3a2
glcak1
aldh9a1ba
aldh9a1bb
aldh2b4
A-Ketoglutaric Acid
glcak1
aldh1a2a
aldh1a2b
D-Xylulose-5-phosphate
UDP-glucose
Arginine and proline metabolism in hepatopancreas and intestine
aldh3a2
aldh9a1ba
aldh9a1bb
odc1a
aoc1b
odc1c
aldh2b4
aldh4a1
agmat
4-Acetamidobutyric Acid
Creatine
L-Ornithine
Spermidine
odc1a
odc1b
zgc:77082
odc1
4-Acetamidobutyric Acid
5-Aminovaleric Acid
Guanidineacetic Acid
N-Acetylputrescine
Spermidine
Urea
Glutathione metabolism in hepatopancreas, intestine, and muscle
pgd
gclm
odc1
ggct
gsto1
odc1
anpep
gsta1
15-Diaminopentane
Cys-Gly
Glutathione Reducedform
L-Cysteine
L-Ornithine
Spermidine
ΓGlutamate-Cysteine
odc1a
odc1b
ggct
odc1c
gsta1
Spermidine
ΓGlutamate-Cysteine
pgd
odc1
gclm
ggct
odc1
gpx1a
15-Diaminopentane
L-Ornithine
ΓGlutamate-Cysteine
PPAR signaling pathway in hepatopancreas
acsbg2
acsl1
pck1
zpr1
acsl3
apoa1
slc27a6
acsbg2
acsl3
cpt1a
acsl4
cpt1a
acsl3
pparaa
pparab
pparγ
LTB4
8-HETE
Pancreatic secretion in hepatopancreas
itpr3
aldh3a2
itpr1
adcy9
prkcb
pla2g12a
rhoac
tmprss9
prkcbb
znf830
slc4a10a
slc4a10b
slc12a2a
slc12a2b
slc12a2c
rab25
kcnma1a
kcnma1b
ca2
Cyclic Amp
